# Discovery of ONO-2920632 (VU6011887): A Highly Selective and CNS Penetrant TREK-2 (TWIK-Related K+ Channel 2) Preferring Activator *In Vivo* Tool Compound

**DOI:** 10.1021/acschemneuro.5c00032

**Published:** 2025-02-21

**Authors:** Kentaro Yashiro, Yuzo Iwaki, Hirohito Urata, Masaya Kokubo, Takahiro Mori, Yoko Sekioka, Koichi Isami, Junya Kato, Joshua Wieting, Kevin M. McGowan, Thomas M. Bridges, Olivier Boutaud, Darren W. Engers, Jerod S. Denton, Haruto Kurata, Craig W. Lindsley

**Affiliations:** † Drug Discovery Chemistry, Ono Pharmaceutical Co., Ltd, 3-1-1 Sakurai, Shimamoto, Mishima, Osaka 618-8585, Japan; ‡ Research Center of Neurology, Ono Pharmaceutical Co., Ltd, 3-1-1 Sakurai, Shimamoto, Mishima, Osaka 618-8585, Japan; § Pharmacokinetic Research, Ono Pharmaceutical Co., Ltd, 3-1-1 Sakurai, Shimamoto, Mishima, Osaka 618-8585, Japan; ∥ Warren Center for Neuroscience Drug Discovery, Vanderbilt University, Nashville, Tennessee 37232, United States; ⊥ Department of Pharmacology, Vanderbilt University School of Medicine, Nashville, Tennessee 37232, United States; # Department of Chemistry, Vanderbilt University, Nashville Tennessee 37232, United States; ∇ Department of Biochemistry, Vanderbilt University, Nashville Tennessee 37232, United States; ○ Department of Anesthesiology, Vanderbilt University Medical Center, Nashville, Tennessee 37232, United States

**Keywords:** TREK (TWIK-Related K+ Channel), K_2_P (two-pore domain potassium channel), pain, ion channel

## Abstract

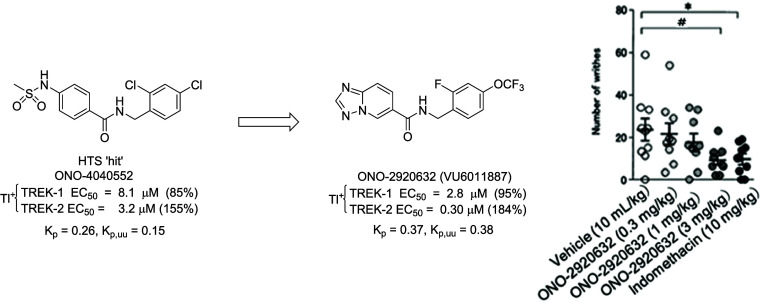

Herein we describe our initial work on the K_2_P family of potassium ion channels with the chemical optimization and characterization of a novel series of TWIK-Related K+ Channel (TREK)-1/2 dual activators and TREK-2 preferring activators derived from a high-throughput screening hit. The exercise provided TREK activators with good CNS penetration and others with low CNS exposure to enable exploration of both central and peripheral TREK activation. From this, ONO-2920632 (VU6011887 = **19b**) emerged as a reasonably potent (human Tl^+^; TREK-1 EC_50_ = 2.8 μM (95% *E*_max_), TREK-2 EC_50_ = 0.30 μM (184% *E*_max_)), first-generation CNS penetrant (rat K_p_ = 0.37) *in vivo* tool compound with selectivity versus the other K_2_P channels (>91-fold selective vs TASK1, TASK2, TASK3, TRAAK, TWIK2, and 31-fold selective vs TRESK) and no significant activity in a large ancillary pharmacology panel. ONO-2920632 (VU6011887) displayed robust, dose dependent efficacy when dosed orally in a mouse pain model (acetic acid writhing assay), where it was equipotent at 3 mg/kg to the assay standard indomethacin at 10 mg/kg. The therapeutic potential of TREK channel activation has long been hampered by a lack of selective, small molecule tools, and this work provides a variety of *in vivo* tool compounds for the community.

## Introduction

The two pore domain (K_2_P) family of potassium channels (encoded by the gene *KCNK*), often referred to as “leak channels”, have garnered a great deal of attention; however, the therapeutic potential of this ion channel family remains obscured by a lack of selective small molecule tools.^1−10^ At present, 15 K_2_P subtypes have been identified within 6 distinct subfamilies: TWIK, TWIK RElated K^+^ channels (TREK), TWIK related Acid-Sensitive K^+^ channels (TASK), TWIK related ALkaline pH-activated K^+^ channels (TALK), Tandem pore domain Halothane Inhibited K^+^ channels (THIK) and TWIK RElated Spinal cord K^+^ channel (TRESK).^1−9^ Of particular interest to our laboratories was the TREK K_2_P subfamily, which consists of TREK-1 (K_2_P2.1), TREK-2 (K_2_P10.1) and TRAAK (K_2_P4.1), and specifically, TREK-1 and TREK-2. Both TREK-1 and TREK-2 are widely expressed in the mammalian CNS, as well as the periphery, and activation of either TREK-1, TREK-2 or both channels have potential therapeutic utility in pain, migraine, ischemia, and arrhythmia among others. TREK-2 is responsible for the background potassium current in primary sensory neurons of the trigeminal ganglia and dorsal root, while TREK-1 is activated downstream of the μ-opioid receptor where it plays a role not in the adverse effects, but the antinociceptive effects of morphine.^10−14^ Thus, selective small molecule activators of TREK-1, TREK-2 or dual TREK-1/2 channels may offer a new opportunity for pain management.

**Figure 1:**
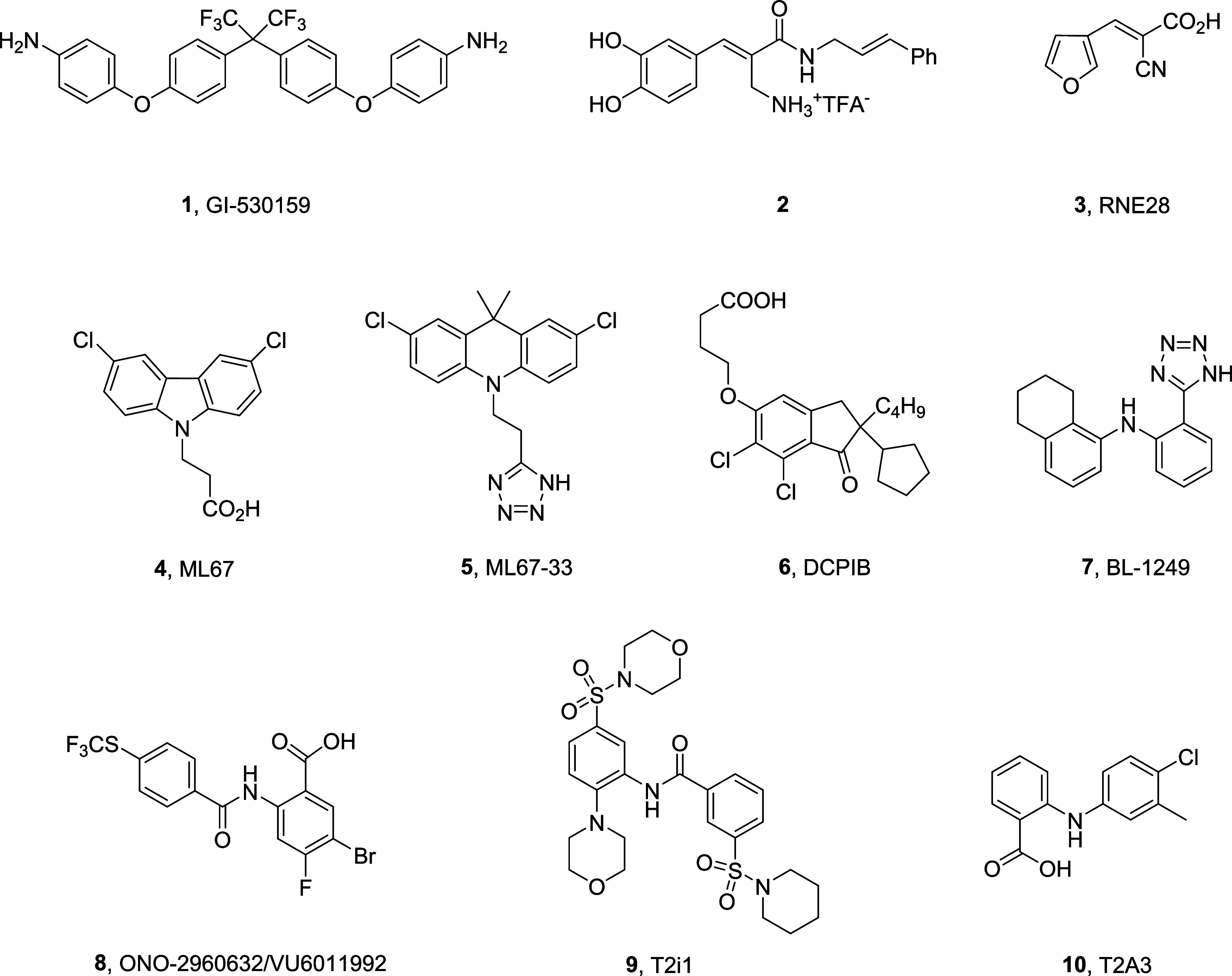
Structures of reported TREK-1 and TREK-2 activators **1**-**10**. To date, these TREK-1/2 ligands lack selectivity and desired potency (mid-to-high μM potency) and/or possess poor physiochemical/drug-like properties coupled with poor DMPK profiles.

Numerous ligands **1**-**10** have been reported to activate the TREK K_2_P family (Figure 1), but the majority lack potency (all mid-to-high μM potency), display poor ion channel selectivity, are electrophilic/reactive, and/or possess poor physiochemical and DMPK properties.^15−22^ When we began our efforts over a decade ago, BL-1249 (**7**), a fenamate class nonsteroidal anti-inflammatory, was an early *in vitro* TREK-1 activator tool compound (actually, a dual TREK-1/2 activator). Attempts at optimization of **7** provided the dual TREK-1/2 activator **8**, which failed to improve functional TREK potency, but did improve unbound fraction and PK.^23^ In 2020, the TREK-1 activator RNE28 (**3**) demonstrated antinociceptive activity in naïve rodents and in models of neuropathic and inflammatory pain, which were blocked by TREK-1 inhibitors and lost in TREK-1 knockout mice.^24^ Importantly, **3** did not induce respiratory depression, rewarding effects, depression, constipation or other morphine-inducing adverse events at efficacious doses; however, the functional EC_50_ of **3** is 37 μM for activation of TREK-1, and pharmacokinetic data were not reported.^24^ Thus, the need for new, *in vivo* tool compounds is essential to further validate TREK-1 and TREK-2 activation as a novel approach for a potentially safer treatment of pain.

## Results and Discussion

### Discovery of TREK Activators

**Figure 2:**
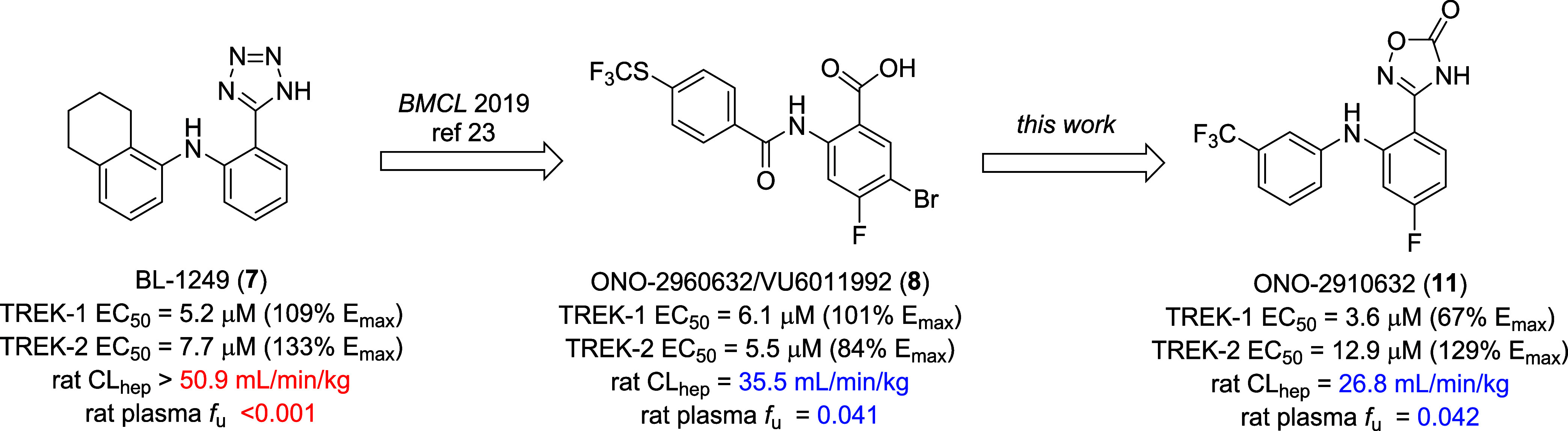
Chemical optimization of BL-1249 (**7**) to afford novel TREK-1/2 dual activators **8** and **11** with comparable potency, but improved rat predicted hepatic clearance and rat plasma protein binding.

We previously reported on optimization efforts focused on the prototypical TREK-1/2 dual activator BL-1249 (**7**), an activator of moderate micromolar potency (TREK-1 EC_50_ = 5.2 ± 1.1 μM, 109 ± 14% *E*_max_; TREK-2 EC_50_ = 7.7 ± 1.9 μM, 133 ± 23% *E*_max_), and high predicted hepatic clearance (rat CL_hep_ > 50.9 mL/min/kg) and exceedingly high (rat plasma *f*_u_ < 0.001) plasma protein binding (Figure 2).^23^ This initial exercise led to the discovery of **8**, a dual TREK activator of comparable functional potency (TREK-1 EC_50_ = 6.1 μM, 101% *E*_max_; TREK-2 EC_50_ = 5.5 μM, 84% *E*_max_), but with improved predicted hepatic clearance (rat CL_hep_ = 35.5 mL/min/kg) and plasma protein binding (rat plasma *f*_u_ = 0.041). Further optimization of **8** led to the development of ONO-2910632 (**11**), wherein the benzoic acid moiety was replaced with a 1,2,4-oxadiazol-5(4*H*)-one bioisostere, affording comparable activity (TREK-1 EC_50_ = 3.6 μM, 67% *E*_max_; TREK-2 EC_50_ = 12.9 μM, 129% *E*_max_) and a comparable *in vitro* DMPK profile (rat CL_hep_ = 26.8 mL/min/kg, rat plasma *f*_u_ = 0.042). After an extensive SAR campaign, it was clear that this chemotype had encountered a potency “floor”, and while physiochemical and DMPK properties could be favorably modulated, TREK-1/2 potency could not broach submicromolar activity.

**Figure 3:**
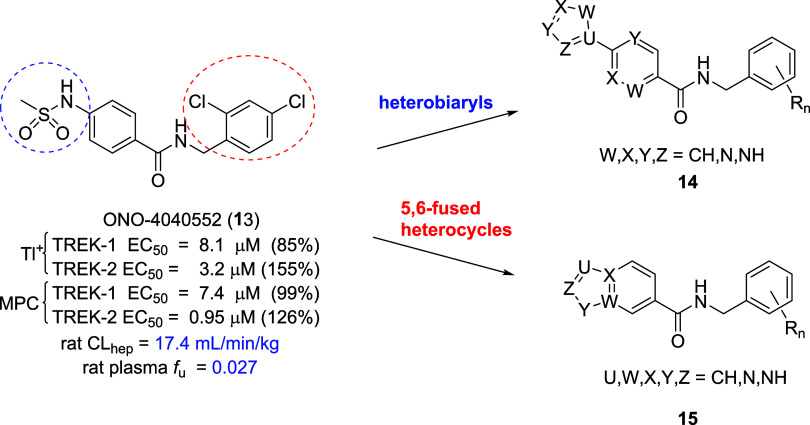
Structure and activities of the HTS hit ONO-4040552 (**13**), a potent TREK-2 preferring activator. Optimization plan focused on alternate amide moieties and either heterobiaryl congeners (**14**) or 5,6-fused heterocycles (**15**) to replace the undesired sulfonamide.

Thus, the team elected to perform a high-throughput screen (approximately 20,000 compounds from the Ono internal library were screened using the IonWorks Barracuda) at Ono utilizing a thallium flux assay to identify fundamentally new chemical matter.^25^ From this effort (Figure 3), a novel TREK-2 preferring activator hit was discovered, ONO-4040552 (**13**, **ML335**), with ∼3-fold selectivity for TREK-2 in the thallium flux assay (TREK-1 EC_50_ = 8.1 μM, 85% *E*_max_; TREK-2 EC_50_ = 3.2 μM, 155% *E*_max_) as well as ∼8-fold selectivity in the follow-up manual patch clamp (MPC) assay (TREK-1 EC_50_ = 7.4 μM, 99% *E*_max_; TREK-2 EC_50_ = 0.95 μM, 126% *E*_max_). Importantly, **13** afforded submicromolar TREK-2 functional potency, and low predicted hepatic clearance (rat CL_hep_ = 17.4 mL/min/kg) and plasma protein binding (rat plasma *f*_u_ = 0.027). Moreover, in an oral rat plasma:brain level study, **13** displayed a *K*_p_ of 0.26 (*K*_p,uu_ of 0.15) and an overall attractive rat PK profile (CL_p_ = 3.5 mL/min/kg, *t*_1/2_ = 3.0 h, *V*_ss_ = 0.787 L/kg). Thus, direct from an HTS screen, the team identified a TREK-2 preferring, submicromolar activator with a good *in vitro* and *in vivo* rat PK profile, as well as being CNS penetrant. However, the *N*-aryl sulfonamide moiety of **13** was deemed unattractive as it could lead to hydrolysis and liberation of a potentially AMES positive aniline, as well as contributing to a modest *K*_p_/*K*_p,uu_. Thus, we envisioned a two-prong approach wherein the sulfonamide moiety would be replaced with either heterobiaryl congeners (**14**) or 5,6-fused heterocyclic motifs (**15**) while simultaneously exploring alternate benzylic derivatives en route to a mouse *in vivo* tool compound.

### Hit-to-Lead for a TREK Activator *In Vivo* Tool Compound

**Scheme 1:**
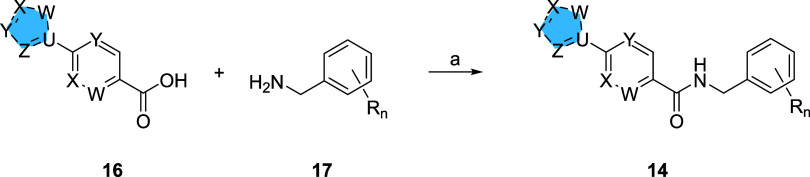
Synthesis of Heterobiaryl Congeners **14** Reagents and conditions: (a) HATU, DIEPA, DMF, rt, 7 h, 43–59%.

For the heterobiaryl analogs **14**, we surveyed a variety of *C*- and *N*-linked five-membered heterocycles to both phenyl and aza-6-membered ring cores employing commercial acids **16** in a HATU-mediated coupling with functionalized benzyl amines **17** to afford amides **14** in yields ranging from 43 to 59% (Scheme 1). SAR for TREK activators proved steep, with little traction in this subseries and few active TREK activators. From this exercise (Table 1), a few interesting examples are presented. A *C*-linked NH-1,2,4-triazole congener **14a** (ONO-2930632, VU6011890) proved to be an ∼ equipotent, dual TREK-1/2 activator in the human thallium assay (TREK-1 EC_50_ = 1.6 μM, 93% *E*_max_; TREK-2 EC_50_ = 0.73 μM, 183% *E*_max_). Similar data was obtained in the follow-up mouse MPC assay (TREK-1 EC_50_ = 1.9 μM, 94% *E*_max_; TREK-2 EC_50_ = 0.61 μM, 97% *E*_max_). In mouse *in vitro* DMPK assays, **14a** showed moderate to high predicted hepatic clearance (CL_hep_ = 33.0 mL/min/kg) and acceptable unbound fraction in plasma (*f*_u_ = 0.022). In a rat cassette PK study, **14a** displayed low clearance (CL_p_ = 20.5 mL/min/kg) a short half-life (*t*_1/2_ = 1.1 h), good volume (*V*_ss_ = 1.5 L/kg) and was centrally penetrant (*K*_p_ = 0.20; *K*_p,uu_ = 0.36). However, we desired a more potent TREK activator to serve as an *in vivo* tool. The analogous *N*-linked 1,2,4-triazole analogs **14b** and **14c** possessed greater TREK activator potency, and they were slightly biased toward TREK-2. Of these, we focused on **14c** (ONO-2950632, VU6012159). In the human thallium flux assay, **14c** was a potent TREK-2 activator (EC_50_ = 0.36 μM, 109% *E*_max_) with low efficacy at TREK-1 (EC_50_ = c.a. 1.1 μM, 20% *E*_max_); moreover, in mouse MPC, **14c** was a potent TREK-2 activator (EC_50_ = 0.31 μM, 72% *E*_max_) with, once again, low efficacy on TREK-1 (EC_50_ = 2.8 μM, 31% *E*_max_), a unique pharmacological profile. In the human ^86^Rb flux K_2_P selectivity panel, **14c** remained a submicromolar TREK-2 activator, a partial TREK-1 activator and >43-fold selective against TASK-1, TASK-2, TASK-3, TRAAK, TWIK-2 and TRESK over TREK-2 (data is not shown). While this was exciting for the program to have such a highly selective and submicromolar TREK-2 activator, the team felt we needed to further improve upon the TREK-2 potency before advancing a compound into mouse POC studies.

**Table 1:**
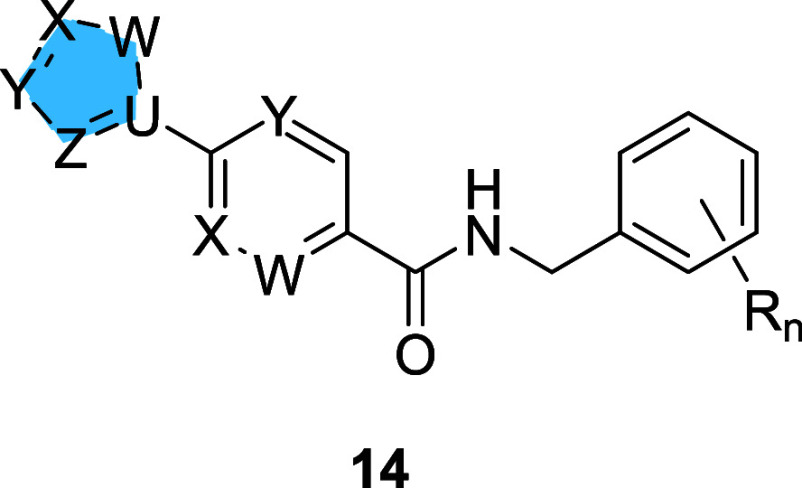

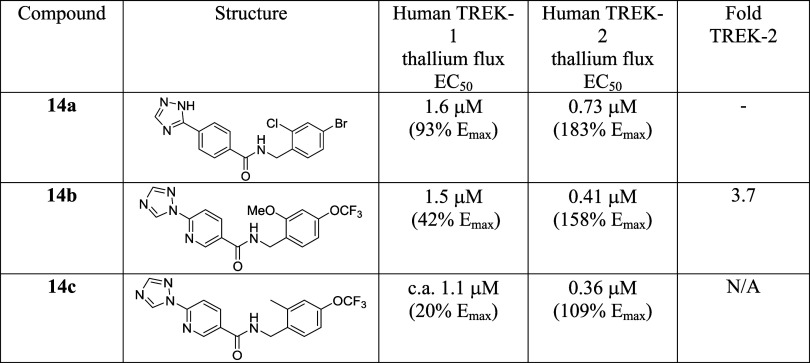
Structures and Activities of Analogs **14^a^**

a Control compound BL-1249: TREK-1 IC_50_ = 5.2 μM (103%); TREK-2 IC_50_ = 7.3 μM (133%).

**Scheme 2:**
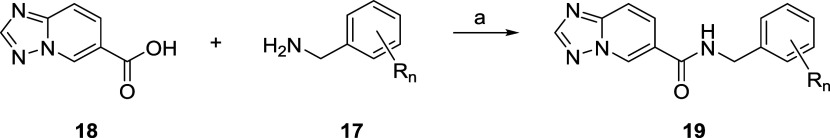
Synthesis of [1,2,4]Triazolo[1,5-*a*]pyridine-Based Benzyl Amides **19** Reagents and conditions: (a) HATU, DIEPA, DMF, rt, 7 h, 68–75%.

After evaluating a number of diverse 5,6-heterobicycles for analogs **15**, a [1,2,4]triazolo[1,5-*a*]pyridine core emerged as a productive replacement for the *N*-arylsulfonamide. Fortunately, the requisite carboxylic acid **18** was commercially available, and coupled with the readily available diversity of benzylic amines **17**, a one-step HATU-mediated amide coupling reaction (Scheme 2) afforded putative TREK-1/2 activators **19** in good overall yields (68–75%). SAR proved steep in this series as well, yet this exercise afforded a few interesting analogs **19a** and **19b** (Table 2) with submicromolar TREK-2 potency in the human thallium flux assay worthy of further examination. The *p*-OCF_3_ moiety on the phenyl ring proved essential for TREK-1/2 activator activity, with both electron-donating and electron-withdrawing *ortho*-substituents tolerated. Of note, **19a** was almost 25-fold TREK-2 preferring, while **19b** was 10-fold TREK-2 preferring. As the goal was a mouse *in vivo* tool compound, we next evaluated these analogs in mouse TREK-1 and TREK-2 MPC assays. Both compounds displayed submicromolar potency on mouse TREK-2 (**19a**: EC_50_ = 0.15 μM, 105% *E*_max_; **19b**: EC_50_ = 0.57 μM, 105% *E*_max_) and micromolar potency on TREK-1 (**19a**: EC_50_ = 5.0 μM, 106% *E*_max_; **19b**: EC_50_ = 2.7 μM, 109% *E*_max_) affording TREK-2 preferences of 33-fold and 4.7-fold, respectively. Both analogs displayed low predicted hepatic clearance and moderate unbound plasma fraction in rat (**19a**: CL_hep_ = 5.7 mL/min/kg, *f*_u_ = 0.075; **19b**; CL_hep_ = 2.9 mL/min/kg, *f*_u_ = 0.075), as well as improved brain penetration in rats 2 h after an oral dose of 3 mg/kg (**19a**: *K*_p_ = 0.50; *K*_p,uu_ = 0.20, **19b**: *K*_p_ = 0.37, *K*_p,uu_ = 0.38) over HTS hit **13** (*K*_p_ = 0.26; *K*_p,uu_ = 0.15). However, in mouse, **19a** showed moderate to high predicted hepatic clearance (CL_hep_ = 30.5 mL/min/kg), while **19b** (CL_hep_ = 17.8 mL/min/kg) was low; in addition, mouse unbound plasma faction was improved for **19b** (*f*_u_ = 0.24) over **19a** (*f*_u_ = 0.10). Based on these data, we elected to further profile **19b** (ONO-2920632, VU6011887).

**Table 2:**
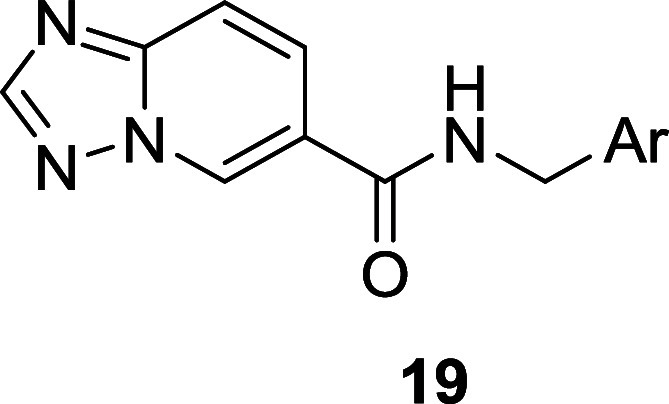

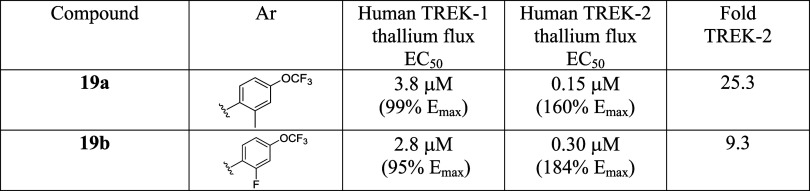
Structures and Activities of Analogs **19^a^**

a Control compound BL-1249: TREK-1 IC_50_ = 5.2 μM (103%); TREK-2 IC_50_ = 7.3 μM (133%).

### Drug Metabolism and Disposition

In a rat PO PK study dosed at 3 mg/kg, **19b** displayed a *C*_max_ of 10.3 μM with a 7.0 h *T*_max_ and *a* > 24 h half-life, mirroring the low predicted hepatic clearance. In this study, total brain concentrations did not diminish between 2 h (2.68 μM) and 24 h (2.89 μM). A similar PK profile was demonstrated in mouse. When **19b** was dosed at 3 mg/kg in C57BL/6 mice, a *C*_max_ of 10.5 μM was noted, along with a 3.8 h *T*_max_ and a 19 h half-life. At the 2-h time point in this study, total brain concentrations averaged 3.2 μM, with 0.92 μM free brain levels (mouse brain *f*_u_ = 0.28) and CSF levels of 0.22 μM. The PK profile extended from rodents to dog, where a low dose IV study (0.1 mg/kg, HP-β-CD solution) afforded excellent PK (CL_p_ = 1.3 mL/min/kg, *t*_1/2_ = 22 h, *V*_ss_ = 2.2 L/kg).

Prior to an *in vivo* proof of concept study in a pain model, we needed to assess broader selectivity among the K_2_P family of ion channels as well as a more comprehensive evaluation of promiscuity in a Eurofins Lead Profiling panel. K_2_P selectivity was performed in a human ^86^Rb Flux assay where **19b** showed submicromolar activity at TREK-2 and micromolar activity at TREK-1, with even greater TREK-2 preference (∼46-fold). In this panel, **19b** was >150-fold selective against TASK-1, TASK-2, TASK-3, TRAAK and TWIK-2 over TREK-2. The only off-target activity in the K_2_P family was at TRESK, where **19b** showed micromolar inhibitor activity (31-fold selective versus TREK-2) (data is not shown). In the Eurofins Lead Profiling panel of 72 GPCRs, ion channels and transporters, there were no displacements of any radioligand >50% at 30 μM, including the three opiate receptors profiled (DOP, 9%@30 μM, KOP, 8%@30 μM and MOP, 1%@30 μM). Thus, the team felt we had a TREK-2 preferring activator **19b** with both the DMPK and selectivity profiles to support the data generated with **3**, and further validate the role of TREK-2 activation in analgesia.

### Analgesic Effect in Acetic Acid Writhing Assay

**Figure 4:**
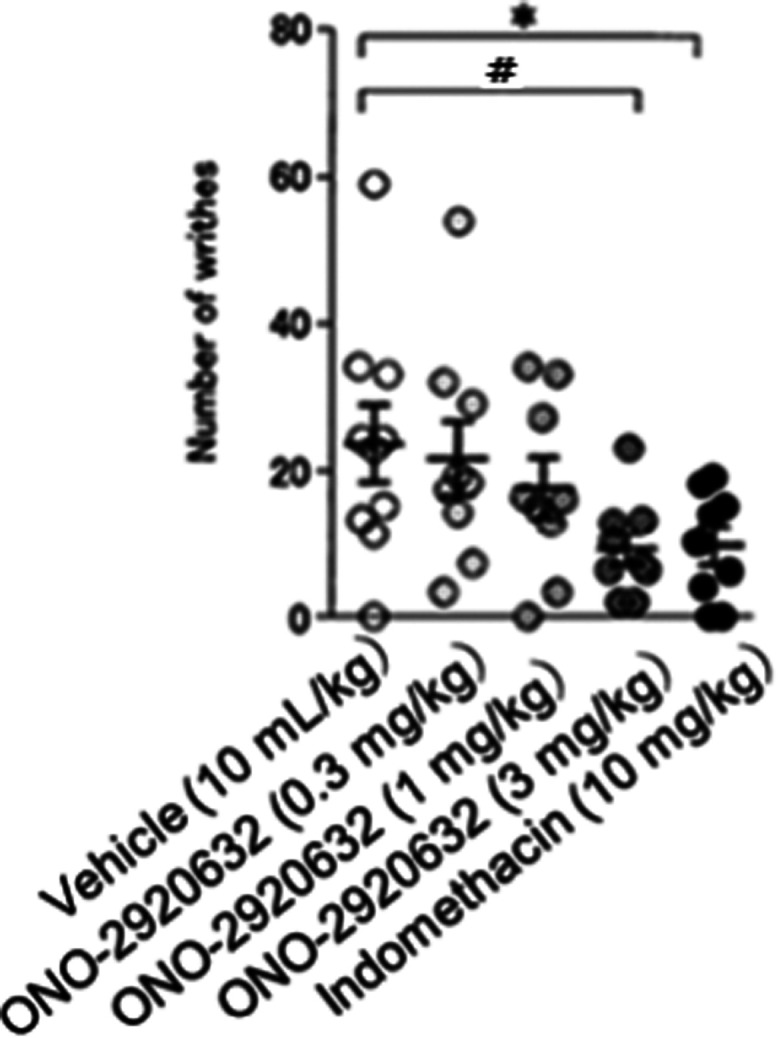
Analgesic effect in acetic acid writhing assay. ICR male mice (6 weeks of age) were pretreated with vehicle (0.5 w/v% Methyl Cellulose in distilled water, PO) or **19b** (= ONO-2920632) (0.3, 1, 3 mg/kg, PO) or Indomethacin (10 mg/kg, PO). Two hours after ONO compound (*n* = 9 per dose group) and vehicle administration, or 1 h after Indomethacin administration (*n* = 10), the animals were injected with acetic acid (0.7% v/v, 10 mL/kg, IP). The number of writhes (characterized by contraction of the abdominal musculature and extension of the limbs) was then counted for 30 min. Analgesic effect was determined by comparing the number of writhes between in the presence of compound and in the presence of vehicle (*; *p* < 0.05 by Student’s *t* test, #; *p* < 0.05 by Williams test).

To initially explore the role of TREK-2 activation in analgesia, we evaluated **19b** in the acetic acid writhing assay. Injection of acetic acid activates nociceptors directly and/or produces inflamed viscera (subdiaphragmatic organs) and subcutaneous (muscle wall) tissues. The number of writhes (characterized by contraction of the abdominal musculature and extension of the limbs) was then counted for 30 min. Analgesic effect was determined by comparing the number of writhes between in the presence of **19b** or vehicle. As shown in Figure 4, when dosed PO, **19b** dose-dependently reduces the number of writhes, with robust efficacy at 3 mg/kg, as compared to the positive control, indomethacin (PO at 10 mg/kg). At the 3 mg/kg PO dose of **19b**, total brain levels are ∼3 μM and free brain in 0.92 μM and CSF levels in 0.22 μM, providing a good PK/PD relationship and supporting the data generated with the TREK activator **3**.

**Figure 5:**
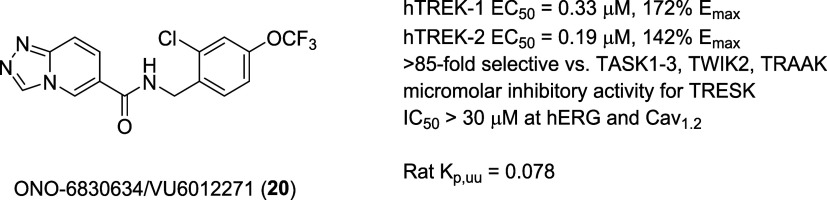
Structure and activities of the peripherally restricted dual TREK-1/2 activator **20**.

Finally, as the goal was to develop TREK activator tool compounds, we also discovered a peripherally restricted dual TREK-1/2 activator **20**, ONO-6830634 (VU6012271) for others to employ to investigate the therapeutic potential of peripheral TREK activation (Figure 5). This tool emerged from the work on analogs **15**, but in this instance harboring a [1,2,4]triazolo[4,3-*b*]pyridine core as opposed to the [1,2,4]triazolo[1,5-*a*]pyridine core of **19b**. Compound **20** proved to be a potent activator of both human TREK-1 (EC_50_ = 0.33 μM, 172% *E*_max_) and human TREK-2 (EC_50_ = 0.19 μM, 142% *E*_max_ in MPC assay), devoid of activity at hERG (IC_50_ > 30 μM in Tl^+^ flux assay) and hCav_1.2_ (IC_50_ > 30 μM in Ca^2+^ flux assay) and with limited CNS exposure in rat (*K*_p,uu_ = 0.078). Importantly, **20** was >91-fold selective in the K_2_P ^86^Rb flux panel against TASK-1, TASK-2, TASK-3, and TWIK-2 over TREK-1/2. The only off-target activity in the K_2_P family was at TRESK, where **20** showed micromolar inhibitor activity and was 85-fold selective against TRAAK over TREK-1 (data is not shown).

## Conclusions

In summary, a thallium flux assay employing TREK-1 and TREK-2 identified an attractive TREK-2 preferring activator **13** with a favorable profile and CNS penetration direct from the HTS screening campaign. The hit-to-lead (hit-to-tool) effort proceeded via an approach wherein the sulfonamide moiety of **13** would be replaced with either heterobiaryl congeners (**14**) or 5,6-fused heterocyclic motifs (**15**). This exercise culminated in the discovery of ONO-2920632/VU6011887 (**19b**) a TREK-2 preferring activator with exceptional selectivity versus the K_2_P ion channel family as well as clean ancillary pharmacology against 72 GPCRs, ion channels and transporters, including the three opiate receptors profiled (DOP, 9%@30 μM, KOP, 8%@30 μM and MOP, 1%@30 μM). Good CNS penetration and an excellent mouse PK profile enabled evaluation of **19b** in the acetic acid writhing assay where it displayed analgesic efficacy at 3 mg/kg PO of comparable magnitude to the positive control indomethacin (10 mg/kg PO). Moreover, there was a PK/PD relationship at the effective dose/exposure. These data support findings from other laboratories on the therapeutic potential of TREK activation for nonopiate pain management. Finally, this work provides the community with best-in-class tool compounds to selectively study TREK activation in the CNS as well as in the periphery with a restricted tool compound.

## Supporting Information Available

Additional experimental details; methods for the synthesis and characterization of all compounds (^1^H NMR, ^13^C NMR, 2-D NMR, HRMS); *in vitro* and *in vivo* DMPK protocols and Supporting Figures (PDF)

## Abbreviations

TREK: TWIK Related K^+^ channels
MED: minimum effective dose
PK: pharmacokinetics
PBL: plasma:brain level study
