# Correction to “Discovery of ONO-2920632 (VU6011887): A Highly Selective and CNS Penetrant TREK‑2 (TWIK-Related K+ Channel 2) Preferring Activator *In Vivo* Tool Compound”

**DOI:** 10.1021/acschemneuro.5c00409

**Published:** 2025-06-16

**Authors:** Kentaro Yashiro, Yuzo Iwaki, Hirohito Urata, Masaya Kokubo, Takahiro Mori, Yoko Sekioka, Koichi Isami, Junya Kato, Joshua Wieting, Kevin M. McGowan, Thomas M. Bridges, Olivier Boutaud, Darren W. Engers, Jerod S. Denton, Haruto Kurata, Craig W. Lindsley

In the original article, there was an error in the structure of compound **20**. The correct structure is given below.
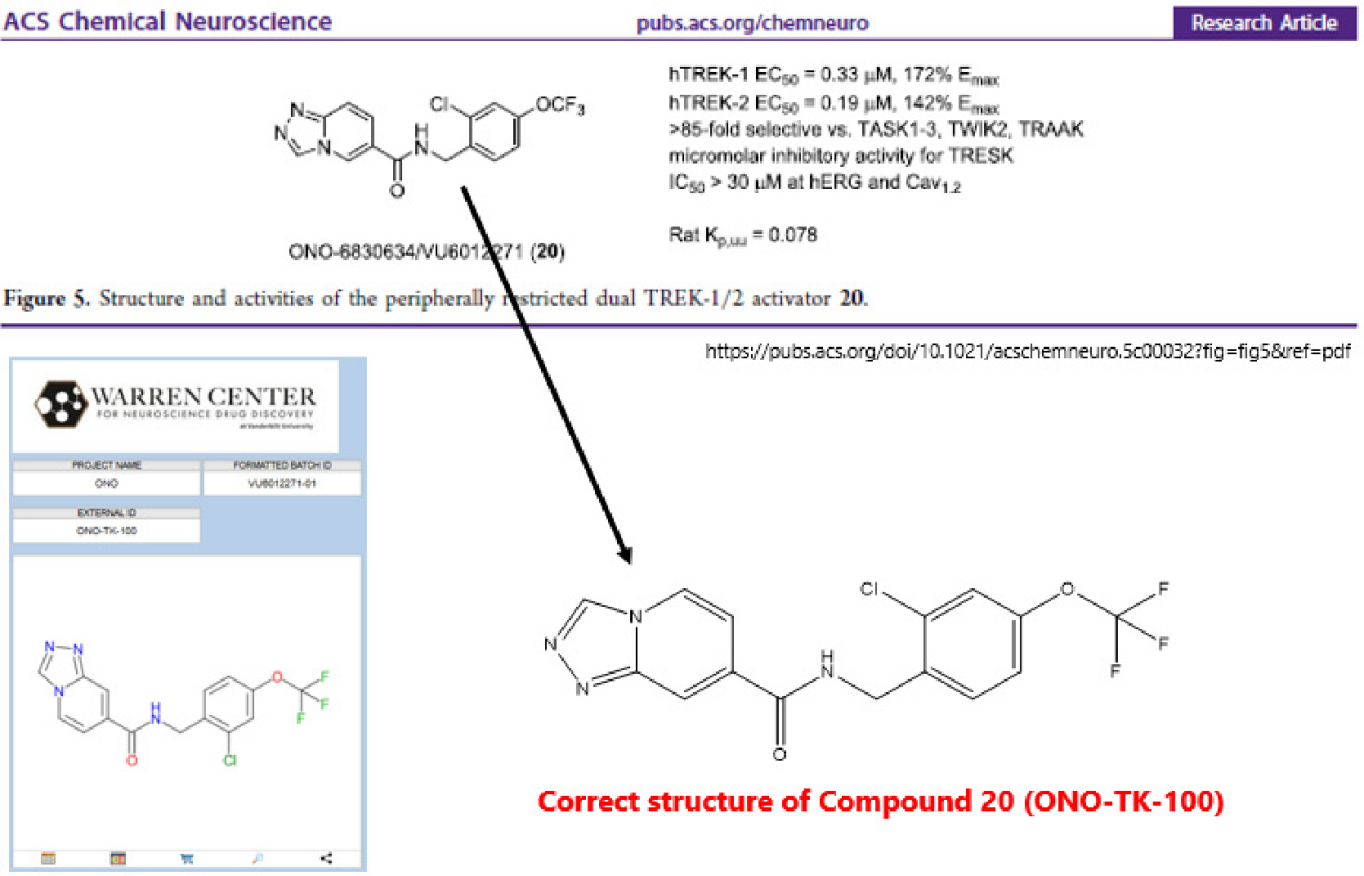


Also the chemical name in the text is modified from “[1,2,4]­triazolo­[**4,3-** *b*]­pyridine core” to “[1,2,4]­triazolo­[**4,3-** *a*]­pyridine core”.

In the original article, there is an error in Figure S5. A corrected version of the Supporting Information has been submitted with the corrected Figure S5.

## Supplementary Material



## Notes

Additional experimental details; methods for the synthesis and characterization of all compounds (^1^H NMR, ^13^C NMR, 2-D NMR, HRMS); *in vitro* and *in vivo* DMPK protocols and Supporting Figures (PDF)

