# Supplementary material for: Correction to “Discovery of ONO-2920632 (VU6011887): A Highly Selective and CNS Penetrant TREK‑2 (TWIK-Related K+ Channel 2) Preferring Activator In Vivo Tool Compound”
Source: ACS Chem Neurosci. 2025 Jun 16;16(13):2559–60. doi: 10.1021/acschemneuro.5c00409 (PMC12226813; doi:10.1021/acschemneuro.5c00409)
Supplement: Supplementary file 1 [file cn5c00409_si_001.pdf]

Discovery of ONO-2920632 (VU6011887): A highly selective and CNS penetrant TREK-2 (TWIK-Related K+ Channel 2) preferring activator *in vivo* tool compound

Kentaro Yashiro,[1] Yuzo Iwaki,[1] Hirohito Urata,[1] Masaya Kokubo,[1] Takahiro Mori,[2] Yoko Sekioka,[2] Koichi Isami,[2] Junya Kato,[3] Joshua Weiting,[4,5] Kevin M. McGowan,[4,5] Thomas M. Bridges,[4,5] Olivier Boutaud,[4,5] Darren W. Engers,[4,5] Jerod S. Denton,[8] Haruto Kurata,*[1] and Craig W. Lindsley*[4,5,6,7]

Affiliation:
[1]Drug Discovery Chemistry, Ono Pharmaceutical Co., Ltd, 3-1-1 Sakurai, Shimamoto, Mishima, Osaka 618-8585, Japan
[2]Research Center of Neurology, Ono Pharmaceutical Co., Ltd, 3-1-1 Sakurai, Shimamoto, Mishima, Osaka 618-8585, Japan
[3]Pharmacokinetic Research, Ono Pharmaceutical Co., Ltd, 3-1-1 Sakurai, Shimamoto, Mishima, Osaka 618-8585, Japan
[4]Warren Center for Neuroscience Drug Discovery, Vanderbilt University, Nashville, TN 37232, USA
[5]Department of Pharmacology, Vanderbilt University School of Medicine, Nashville, TN 37232, USA
[6]Department of Chemistry, Vanderbilt University, Nashville TN 37232, USA
[7]Department of Biochemistry, Vanderbilt University, Nashville TN 37232, USA
[8]Department of Anesthesiology, Vanderbilt University Medical Center, Nashville, TN 37232, USA

*To whom correspondence should be addressed at craig.lindsley@vanderbilt.edu, h.kurata@ono-pharma.com

TABLE OF CONTENTS

Eurofins Lead Profiling Screen....................S2
Procedures for Biological Experiments....................S4
General Methods....................S10
Synthetic Procedures and spectra....................S12
Supplemental Figures....................S21

Table S1. Eurofins Lead Profiling Screen Data

This is a radioligand binding panel of 72 targets including GPCRs, ion channels, transporters and nuclear hormones. Biochemical assay results are presented as the percent inhibition of specific binding at a 30 μM concentration of **ONO-2920632 (VU6011887,** Compound **19b**).

**Target/Protein**	**Species**	**% Inhibition at 10 μM**
Adenosine A_1	Human	14
Adenosine A_{2A}	Human	10
Adenosine A_3	Human	21
Adrenergic α_{1A}	Rat	2
Adrenergic α_{1B}	Rat	6
Adrenergic α_{1D}	Human	4
Adrenergic α_{2A}	Human	3
Adrenergic β_1	Human	9
Adrenergic β_2	Human	2
Androgen (Testosterone)	Human	18
Bradykinin B_1	Human	-12
Bradykinin B_2	Human	5
Calcium Channel L-Type, Benzothiazepine	Rat	2
Calcium Channel L-Type, Dihydropyridine	Rat	-2
Calcium Channel N-Type	Rat	-1
Cannabinoid CB_1	Human	17
Dopamine D_1	Human	22
Dopamine D_{2S}	Human	6
Dopamine D_3	Human	13
Dopamine $D_{4.2}$	Human	15
Endothelin ET_A	Human	-3
Endothelin ET_B	Human	4
Epidermal Growth Factor (EGF)	Human	2
Estrogen ERα	Human	1
$GABA_A$, Flunitrazepam, Central	Rat	0
$GABA_A$, Muscimol, Central	Rat	-1
$GABA_{B1A}$	Human	-1
Glucocorticoid	Human	7
Glutamate, Kainate	Rat	13
Glutamate, NMDA, Agonism	Rat	-3
Glutamate, NMDA, Glycine	Rat	-1
Glutamate, NMDA, Phencyclidine	Rat	0
Glycine, Strychnine-Sensitive	Rat	-16
Histamine H_1	Human	8

Histamine H_2	Human	-20
Histamine H_3	Human	1
Imidazoline I_2, Central	Rat	2
Interleukin IL-1 R1	Mouse	9
Leukotriene, Cysteinyl $CysLT_1$	Human	5
Melatonin MT_1	Human	17
Muscarinic M_1	Human	1
Muscarinic M_2	Human	-3
Muscarinic M_3	Human	8
Neuropeptide Y Y_1	Human	2
Neuropeptide Y Y_2	Human	-1
Nicotinic Acetylcholine α3β4	Human	-17
Nicotinic Acetylcholine α1, Bungarotoxin	Human	20
Opiate δ_1 (OP1, DOP)	Human	9
Opiate κ (OP2, KOP)	Human	8
Opiate μ (OP3, MOP)	Human	1
Phorbol Ester	Mouse	0
Platelet Activating Factor (PAF)	Human	12
Potassium Channel [K_{ATP}]	Hamster	13
Potassium Channel [SK_{CA}]	Rat	4
Potassium Channel hERG	Human	44
Prostanoid EP_4	Human	14
Purinergic P2X	Rabbit	22
Purinergic P2Y, Non-Selective	Rat	-7
Rolipram	Rat	13
Ryanodine	Rat	6
Serotonin (5-HT_{1A})	Human	2
Serotonin (5-HT_{2B})	Human	14
Serotonin (5-HT_3)	Human	4
Sigma σ_1	Human	45
Sodium Channel, Site 2	Rat	15
Tachykinin NK_1	Human	2
Thyroid Hormone	Rat	9
Transporter, Dopamine (DAT)	Human	14
Transporter, GABA	Rat	-6
Transporter, Norepinephrine (NET)	Human	29
Transporter, Serotonin (SERT)	Human	2
Vanilloid	Rat	-8

Procedures for Biological Experiments

TREK-1 and TREK-2 thallium flux assay protocol

CHO cells stably expressing human TREK-1 (hTREK-1/CHO) were constructed at Evotec. HEK293 cells stably expressing human TREK-2 (hTREK-2/HEK) were constructed at Ono. hTREK-1/CHO or hTREK-2/HEK were plated in 384-well plates at a density of 1.0×10^4 cells/well or 2.0×10^4 cells/well, respectively, and cultured overnight. After the wash by HBSS + 20 mM HEPES buffer, cells were loaded with Thallos dye for 1 hour at room temperature. After the wash by HBSS + 20mM HEPES buffer, test compounds, control compound (BL-1249) or 0.3% DMSO dissolved with HBSS + 20mM HEPES buffer were added to each well. After 10 minutes incubation at room temperature, thallium stimulus buffer were added to each well and the fluorescent intensity are measured by Panoptic. The final thallium concentration was 0.36 mM in TREK-1 or 0.24 mM in TREK-2, respectively. The change of fluorescent intensity (ΔRatio) and % activation to compare the efficacy and potency of test compounds were calculated using the following equations.

Δ Ratio = (fluorescent intensity at 25 seconds after thallium addition)/(average of fluorescent intensity at pre-read)

% activation = (ΔRatio of test compound - ΔRatio of 0.3% DMSO)/(ΔRatio of 10 μM control compound - ΔRatio of 0.3% DMSO)

hERG thallium flux assay protocol

HEK293 cells stably expressing human ERG (hERG/HEK) were kindly provided by Dr. Weaver, Vanderbilt University Medical Center. hERG/HEK were plated in 384-well plates at a density of 2.0×10^4 cells/well, and cultured overnight. After the wash by HBSS + 20 mM HEPES buffer, cells were loaded with Thallos dye for 1 hour at room temperature. After the wash by HBSS + 20mM HEPES buffer, test compounds, control compounds (dofetilide) or 0.3% DMSO dissolved with 90 mM K+ buffer were added to each well. The final K+ concentration was 45 mM. After 10 minutes incubation at room temperature, thallium stimulus buffer were added to each well and the fluorescent intensity are measured by Panoptic. The final thallium concentration was 0.96 mM. The change of fluorescent intensity (ΔRatio) and % inhibition to compare the efficacy and potency of test compounds were calculated using the following equations.

Δ Ratio = (fluorescent intensity at 25 seconds after thallium addition)/(average of fluorescent intensity at pre-read)

% inhibition = 100 - (ΔRatio of test compound - ΔRatio of 1 μM control compound)/(ΔRatio of 0.3% DMSO - ΔRatio of 1 μM control compound)x100

$Ca_{v1.2}$ calcium flux assay protocol

CHO cells stably expressing human Cav1.2/b2/a2/d1 hCav1.2/CHO were purchased from ChanTest. hCav1.2/CHO were plated in 384-well plates at a density of $1.0x10^4$ cells/well, and cultured overnight. After the wash by HBSS + 20 mM HEPES buffer, cells were loaded with Fluo-4 dye for 1 hour at room temperature. After the wash by HBSS + 20mM HEPES buffer, the fluorescent intensity was measured by Panoptic and test compounds, control compounds (verapamil) or 0.3% DMSO were added to each well. The change of fluorescent intensity (ΔRatio) and % inhibition to compare the efficacy and potency of test compounds were calculated using the following equations.

Δ Ratio = (fluorescent intensity at 300 seconds after compound addition)/(average of fluorescent intensity at pre-read)

% inhibition = 100 - (ΔRatio of test compound - ΔRatio of 10 μM control compound)/(ΔRatio of 0.3% DMSO - ΔRatio of 10 μM control compound)x100

Drug Metabolism Methods:

***In vitro* Plasma protein binding and Brain homogenate binding:** Determination of fraction unbound (f_u) in plasma was conducted in vitro via equilibrium dialysis using HTDialysis (HTD) membrane plates. The top half of the plate was filled with 100 uL of Dubelco's Phosphate Buffered Saline, pH 7.4 (DPBS). Compounds were diluted into plasma from each species (5 μM final concentration), which was aliquoted in triplicate to the 'bottom half' of the prepared HTD plate wells. The HTD plate was sealed and incubated for 6 hours at 37 ˚C. Following incubation, each well (both top and bottom halves) were transferred (20 μL) to the corresponding wells of a 96-shallow-well (V-bottom) plate. The daughter plates were then matrix-matched (DPBS side wells received equal volume of plasma, and plasma side wells received equal volume of DPBS), and extraction solution (120 μL; acetonitrile containing 50 nM carbamazepine as IS) was added to all wells of both daughter plates to precipitate protein and extract test article. The plates were then sealed and centrifuged (3500 rcf) for 10 minutes at ambient temperature. Supernatant (60 μL) from each well of the daughter plates was then transferred to the corresponding wells of new

daughter plates (96-shallow-well, V bottom) containing water (Milli-Q, 60 µL/well), and the plates were sealed in preparation for LC-MS/MS analysis (see LC-MS/MS analysis method below).

The unbound fraction (f_u) was calculated following the equation below, and mean values for each species were calculated from 3 replicates.

A similar approach was used to determine the degree of brain homogenate binding, which employed the same methodology and procedure with the following modifications: 1) a final compound concentration of 1 µM was used, 2) naïve rat brains were homogenized in DPBS (1:3 composition of brain: DPBS, w/w) using a Mini-Bead Beater™ machine in order to obtain brain homogenate, which was then treated in the same manner as the plasma samples in the previously described plasma protein binding assay. Fraction unbound for both plasma and brain samples was determined using Equation 4.

$$f_u = \frac{Conc_{buffer}}{Conc_{plasma}}$$

Equation 4 Determination of fraction unbound in plasma.

The diluted fraction unbound (f_u2) in brain was calculated in the same manner by using brain homogenate rather than plasma. Undiluted fraction unbound for the brain was calculated using Equation 5

$$f_u = \frac{1/4}{\left\{\left(\frac{1}{fu2}\right) - 1\right\} + 1/4}$$

Equation 5 Determination of fraction unbound in brain. F_u2 represents the diluted fraction unbound.

Intrinsic clearance: Human or rat hepatic microsomes (0.5 mg/mL) and 1 µM test compound were incubated in 100 mM potassium phosphate pH 7.4 buffer with 3 mM $MgCl_2$ at 37 °C with constant shaking. After a 5 min preincubation, the reaction was initiated by the addition of NADPH (1 mM). At selected time intervals (0, 3, 7, 15, 25, and 45 min), aliquots were taken and subsequently placed into a 96-well plate containing cold acetonitrile with internal standard (50 ng/mL carbamazepine). Plates were then centrifuged at 3000 rcf (4 °C) for 10 min, and the supernatant was transferred to a separate 96-well plate and diluted 1:1 with water for LC/MS/MS analysis. The *in vitro* half-life ($_{1/2}$, min, Eq. 1), intrinsic clearance (CL_{int}, mL/min/kg, Eq. 2), and

subsequent predicted hepatic clearance (CL_{hep}, mL/min/kg, Eq. 3) was determined employing the following equations:

$$(1)\ T_{1/2} = \frac{Ln(2)}{K}$$

where k represents the slope from linear regression analysis of the natural log percent remaining of a test compound as a function of incubation time

$$(2)\ CL_{int} = \frac{0.693}{in\ vitro T_{1/2}} x \frac{mL\ incubation}{mg\ microsomes} x \frac{45\ mg\ microsomes}{gram\ liver} x \frac{20^{a}\ gram\ liver}{kg\ body\ wt}$$

[a]scale-up factors: of 20 (human) or 45 (rat)

$$(3)\ CL_{hep} = \frac{Q_h \cdot CL_{int}}{Q_h + CL_{int}}$$

where Q_h (hepatic blood flow, mL/min/kg) is 21 (human) or 70 (rat).

LC/MS/MS Bioanalysis of Samples from Plasma Protein Binding and Intrinsic Clearance Assays:

Samples were analyzed on a Thermo Electron TSQ Quantum Ultra triple quad mass spectrometer (San Jose, CA) via electrospray ionization (ESI) with two Themo Electron Accella pumps (San Jose, CA), and a Leap Technologies CTC PAL autosampler (Carrboro, NC). Analytes were separated by gradient elution on a dual column system with two Thermo Hypersil Gold (2.1 x 30 mm, 1.9 µm) columns (San Jose, CA) thermostated at 40 °C. HPLC mobile phase A was 0.1% formic acid in water and mobile phase B was 0.1% formic acid in acetonitrile. The gradient started at 10% B after a 0.2 min hold and was linearly increased to 95% B over 0.8 min; hold at 95% B for 0.2 min; returned to 10% B in 0.1 min. The total run time was 1.3 min and the HPLC flow rate was 0.8 mL/min. While pump 1 ran the gradient method, pump 2 equilibrated the alternate column isocratically at 10% B. Compound optimization, data collection, and processing was performed using Thermo Electron's QuickQuan software (v2.3) and Xcalibur (v2.0.7 SP1).

***In vivo* DMPK experimental:**

Determination of brain to plasma ratio:

Animal care and use

All animal study procedures were approved by the Institutional Animal Care and Use Committee and were conducted in accordance with the National Institutes of Health regulations of animal care covered in Principles of Laboratory Animal Care (National Institutes of Health). All rats were fasted overnight prior to testing.

In-life phase

For determination of the brain over plasma ratio (K_p), compounds were formulated in 8% ethanol, 32% PEG400 and 60% DMSO (*v/v/v*) and administered as a single 0.2 mg/kg IV dose (1 mL/kg) to male, Sprague Dawley rats ($n = 1$) via injection into a surgically-implanted jugular vein catheter. At 15 min post dosing, blood sample was collected into chilled, K_2EDTA anticoagulant-fortified tube and immediately placed on wet ice. The blood sample was then centrifuged (1700 rcf, 5 minutes, 4 ˚C) to obtain plasma sample. At the same post-administration time point, whole brain sample was obtained by rapid dissection, rinsed with PBS, and immediately frozen in individual tissue collection box (dry ice). All brain and plasma samples were stored at -80 ˚C until analysis by LC-MS/MS.

Sample Analysis: Concentrations in plasma and brain homogenates were quantified by liquid chromatography tandem mass spectrometry (LC-MS/MS). Whole brains were homogenized in 3 mL of 70:30 IPA:water in a mini bead beater for 3 min, and centrifuged at 3,500 g for 5 min. 5 uL of the supernatant was diluted in 15 uL of blank plasma for quantification of the analytes. Plasma samples were centrifuged at 3,500 g for 5 min. A standard curve was generated by diluting the analytes DMSO stocks with blank plasma to obtain a final concentration of 10,000 ng/ml followed by a serial dilution down to 0.5 ng/ml. Quality controls were generated by a serial dilution of the 5,000 ng/ml standard curve solution in blank plasma to obtain 3 concentrations of 500, 50, and 5 ng/ml. 20 uL of brain diluted in plasma, plasma, blank plasma, standard curve and QC samples were loaded in a V-bottom 96-well plate. 120 uL of acetonitrile containing 0.05 uM carbamazepine (internal standard) was added to each well and the plate was centrifuged at 3,500 g for 5 min. 60 uL of the supernatant of each well (protein free) was transferred to a new 96-well plate containing 60 uL of water. The plates were sealed for analysis by LC-MS/MS.

Plasma and brain tissue samples originating from *in vivo* studies were analyzed by electrospray ionization using an AB Sciex Q-TRAP 5500 (Foster City, CA) that was coupled to a Shimadzu LC-20AD pump (Columbia, MD) and a Leap Technologies CTC PAL auto-sampler (Carrboro, NC). Analytes were separated by gradient elution using a C18 column (3 x 50 mm, 3 mm; Fortis Technologies Ltd, Cheshire, UK) that was thermostated at 40 °C. HPLC mobile phase A was 0.1% formic acid in water (pH unadjusted); mobile phase B was 0.1% formic acid in acetonitrile (pH unadjusted). A 10% B gradient was held for 0.2 min and was linearly increased to 90% B over 0.8 min, with an isocratic hold for 0.5 min, before transitioning to 10% B over 0.05 min. The column was re-equilibrated (1 min) before the next sample injection. The total run time was 2.55 min, and the HPLC flow rate was 0.5 ml/min. The source temperature was set at 500 °C, and mass spectral analyses were performed using a Turbo-Ion spray source in positive ionization mode (5.0-kV spray voltage) and using multiple-reaction monitoring of transitions specific for the analytes. All data were analyzed using AB Sciex Analyst 1.5.1 software.

Brain plasma concentration ratio (K_p) was calculated by dividing brain concentration by plasma concentration for each animal. Unbound brain to unbound plasma concentration ratio ($K_{p,uu}$) is calculated using the following formula: $K_{p,uu}$ = (Brain ng/g x brain fu)/(plasma ng/ml x plasma fu).

For determination of the plasma concentration-time profile, compounds were formulated in methyl cellulose and administered as a 3 mg/kg PO dose to male C57BL/6 mouse or SD rat (n = 2). For the same determination, compounds were formulated in HP-β-CD and administered as a 0.1 mg/kg IV dose to male beagle dog (n = 2).

Plasma samples originating from *in vivo* studies were analyzed by electrospray ionization using a Q-TRAP 5500 (AB Sciex) that was coupled to a Prominence UFLC XR (Shimadzu Corporation). Analytes were separated by gradient elution using a Shim-pack XR-ODS II (2 x 75 mm, 2.2µm, Shimadzu Corporation) that was thermostated at 40 °C. HPLC mobile phase A was 5 mM ammonium acetate containing 0.2% formic acid in water (pH unadjusted); mobile phase B was acetonitrile. A 10% B gradient was linearly increased to 90% B over 1.5 min, with an isocratic hold for 1.5 min, before transitioning to 10% B over 0.1 min. The column was re-equilibrated (0.9 min) before the next sample injection. The total run time was 4.0 min, and the HPLC flow rate was 0.5 ml/min. The source temperature was set at 600 °C, and mass spectral analyses were performed using a Turbo-Ion spray source in positive ionization mode and using multiple-reaction

monitoring of transitions specific for the analytes. All data were analyzed using AB Sciex Analyst software.

Experimental for the analgesic effect in acetic acid writhing assay.

ICR mice were pretreated with vehicle (0.5w/v% Methyl Cellulose in distilled water, p.o.) or ONO-2920632 (0.3, 1, 3 mg/kg, p.o.) or Indomethacin (10 mg/kg, p.o.). **2 hours after ONO compound and vehicle administration**, or 1 hour after Indomethacin administration, the animals were injected with acetic acid (0.7% v/v, 10 mL/kg, i.p.). This chemical activates nociceptors directly and/or produces inflamed viscera (subdiaphragmatic organs) and subcutaneous (muscle wall) tissues. The number of writhes (characterized by contraction of the abdominal musculature and extension of the limbs) **was then counted for 30 min**. Analgesic effect was determined by comparing the number of writhes between in the presence of compound and in the presence of vehicle.

General Methods

All NMR spectra were recorded on a 400 MHz AMX Bruker NMR spectrometer or a 600 MHz VNS600 Agilent NMR spectrometer. ^{1}H and ^{13}C chemical shifts are reported in δ values in ppm downfield with the deuterated solvent as the internal standard. Data are reported as follows: chemical shift, multiplicity (s = singlet, d = doublet, t = triplet, q = quartet, b = broad, m = multiplet), integration, coupling constant (Hz). Low resolution mass spectra were obtained on an Agilent 6120/6150 or Waters QDa (Performance) SQ MS with ESI source. *Method A (Agilent 6120/6150):* MS parameters were as follows: fragmentor: 70, capillary voltage: 3000 V, nebulizer pressure: 30 psig, drying gas flow: 13 L/min, drying gas temperature: 350 ºC. Samples were introduced via an Agilent 1290 UHPLC comprised of a G4220A binary pump, G4226A ALS, G1316C TCC, and G4212A DAD with ULD flow cell. UV absorption was generally observed at 215 nm and 254 nm with a 4 nm bandwidth. Column: Waters Acquity BEH C18, 1.0 x 50 mm, 1.7 um. Gradient conditions: 5% to 95% CH_3CN in H_2O (0.1% TFA) over 1.4 min, hold at 95% CH_3CN for 0.1 min, 0.5 mL/min, 55 ºC. *Method B (Agilent 6120/6150):* MS parameters were as follows: fragmentor: 100, capillary voltage: 3000 V, nebulizer pressure: 40 psig, drying gas flow:

11 L/min, drying gas temperature: 350 ºC. Samples were introduced via an Agilent 1200 HPLC comprised of a degasser, G1312A binary pump, G1367B HP-ALS, G1316A TCC, G1315D DAD, and a Varian 380 ELSD (if applicable). UV absorption was generally observed at 215 nm and 254 nm with a 4 nm bandwidth. Column: Thermo Accucore C18, 2.1 x 30 mm, 2.6 um. Gradient conditions: 7% to 95% CH_3CN in H_2O (0.1% TFA) over 1.6 min, hold at 95% CH_3CN for 0.35 min, 1.5 mL/min, 45 ºC. *Method C (Waters QDa (Performance) SQ MS)*: MS parameters were as follows: cone voltage: 15 V, capillary voltage: 0.8 kV, probe temperature: 600 °C. Samples were introduced via an Acquity I-Class PLUS UPLC comprised of a BSM, FL-SM, CH-A, and PDA. UV absorption was generally observed at 215 nm and 254 nm; 4 nm bandwidth. Column: Phenomenex EVO C18, 1.0 x 50 mm, 1.7 um. Column temperature: 55 °C. Flow rate: 0.4 mL/min. Default gradient: 5% to 95% CH_3CN (0.05% TFA) in H_2O (0.05% TFA) over 1.4 min (curve 6), hold at 95% CH_3CN for 0.1 min. "Polar" (2% to 70% CH_3CN (0.05% TFA) in H_2O (0.05% TFA) over 0.8 min (curve 6), transition to 95% CH_3CN over 0.1 min (curve 6), hold at 95% CH_3CN for 0.6 min.) and "Non-Polar" (40% to 95% CH_3CN (0.05% TFA) in H_2O (0.05% TFA) over 1.4 min (curve 6), hold at 95% CH_3CN for 0.1 min.) gradients were also available. *Method D (Waters QDa (Performance) SQ MS):* MS parameters were as follows: cone voltage: 15 V, capillary voltage: 0.8 kV, probe temperature: 600 °C. Samples were introduced via an Acquity I-Class PLUS UPLC comprised of a BSM, FL-SM, CH-A, and PDA. UV absorption was generally observed at 215 nm and 254 nm with a 4 nm bandwidth. Column: Phenomenex EVO C18, 1.0 x 50 mm, 1.7 um. Column temperature: 55° C. Flow rate: 0.4 mL/min. Default gradient: 5% to 95% CH_3CN in H_2O (5 mM NH_4HCO_3) over 1.4 min (curve 6), hold at 95% CH_3CN for 0.1 min. "Polar" (2% to 70% CH_3CN in H_2O (5 mM NH_4HCO_3) over 0.8 min (curve 6), transition to 95% CH_3CN over 0.1 min (curve 6), hold at 95% CH_3CN for 0.6 min.) and "Non-Polar" (40% to 95% CH_3CN in H_2O (5 mM NH_4HCO_3) over 1.4 min (curve 6), hold at 95% CH_3CN for 0.1 min.) gradients were also available. High resolution mass spectra were obtained on a Thermo Fisher Scientific LTQ Orbitrap XL with ESI source. MS parameters were as follows: Capillary Temp: 340 ºC, Sheath Gas Flow: 55, Aux Gas Flow: 15, Positive Polarity Source Voltage: 4500 V, Source Current: 100 µA, Capillary Voltage: 30 V, Tube Lens: 70 V. Samples were introduced via an Shimadzu UFLCXR HPLC. UV absorption was observed at DAD (190-400nm). Column: Imtakt Unison UK-C18, 3 µm, 2 x 100 mm. Gradient conditions: 10% to 90% CH_3CN (0.1% formic acid) over 10 min, 0.3 mL/min, 40 ºC . For compounds that were purified on a Gilson

preparative reversed-phase HPLC, the system comprised of a 333 aqueous pump with solvent selection valve, 334 organic pump, GX 271 or GX-281 liquid hander, two column switching valves, and a 155 UV detector. UV wavelength for fraction collection was user-defined, with absorbance at 254 nm always monitored. Method 1: Phenomenex Axia-packed Luna C18, 30 x 50 mm, 5 µm column. Mobile phase: CH_3CN in H_2O (0.1% TFA). Gradient conditions: 0.75 min equilibration, followed by user defined gradient (starting organic percentage, ending organic percentage, duration), hold at 95% CH_3CN in H_2O (0.1% TFA) for 1 min, 50 mL/min, 23 ºC. Method 2: Phenomenex Axia packed Gemini C18, 50 x 250 mm, 10 um column. Mobile phase: CH_3CN in H_2O (0.1% TFA). Gradient conditions: 7 min equilibration, followed by user defined gradient (starting organic percentage, ending organic percentage, duration), hold at 95% CH_3CN in H_2O (0.1% TFA) for 7 min, 120 mL/min, 23 ºC. Solvents for extraction, washing and chromatography were HPLC grade. All compounds are >95% purity by HPLC.

LCMS Method-1

Low resolution mass spectra were obtained on an Agilent 6120 or 6150 with ESI source. MS parameters were as follows: fragmentor: 70, capillary voltage: 3000 V, nebulizer pressure: 30 psig, drying gas flow: 13 L/min, drying gas temperature: 350°C. Samples were introduced via an Agilent 1290 UHPLC comprised of a G4220A binary pump, G4226A ALS, G1316C TCC, and G4212A DAD with ULD flow cell. UV absorption was generally observed at 215 nm and 254 nm with a 4 nm bandwidth. Column: Waters Acquity BEH C18, 1.0 mm x 50 mm, 1.7 µm. Gradient conditions: 5% to 95% CH_3CN in H_2O (0.1% TFA) over 1.4 min, hold at 95% CH_3CN for 0.1 min, 0.5 mL/min, 55°C.

LCMS Method-2

Low resolution mass spectra were obtained on a Waters Acquity UPLC I-Class or H-Class system with photodiode array (PDA) detector, MS and ELSD via the following conditions. Column: YMC Triart C18 2.0 mm x 30 mm, 1.9 µm. Mobile phase A: 0.10% TFA in water (v/v). Mobile phase B: 0.10% TFA in MeCN (v/v). Gradient: 95.0% water/5.0% MeCN linear to 5% water/95% MeCN in 1.2 min, HOLD at 5% water/95% MeCN to 1.5 min. Flow: 1.0 mL/min.

Chemical Synthesis.

General Procedures

To a screw-capped vial equipped with a magnetic stir bar were added a carboxylic acid, HATU (1 equiv.), *N,N*-diisopropylethylamine (2 equiv.) and DMF (0.5 - 0.6 mol/L), followed by a solution of an amine (1 equiv.) in DMF (0.6 mol/L). This mixture was allowed to stir at room temperature for 7 hours. The reaction mixture was diluted with saturated aqueous sodium bicarbonate solution and extracted with EtOAc. The combined organic extracts were concentrated under a stream of air. The crude residue was purified by reverse-phase HPLC (eluting with 0.1% trifluoroacetic acid and acetonitrile) to afford the amide compound.

Compound 14c ((ONO-2950632, VU6012159), *N*-(2-methyl-4-(trifluoromethoxy)benzyl)-6-(1H-1,2,4-triazol-1-yl)nicotinamide) was prepared according to the general procedures above using 6-(1*H*-1,2,4-triazol-1-yl)nicotinic acid (100 mg, 0.53 mmol), HATU, *N,N*-diisopropylethylamine, (2-methyl-4-(trifluoromethoxy)phenyl)methanamine and DMF in 29% yield (57 mg). LCMS (Method 2): Retention Time = 0.93 min; m/z $(M + 1)^+$ = 378. HRMS: Obs: 378.1167, Calcd: 378.1172 for $C_{17}H_{15}F_3N_5O_2$. ^{1}H NMR (600 MHz, d_6-DMSO) δ 9.45 (s, 1H), 9.24 (br t, J=5.6 Hz, 1H), 8.99 (d, J=2.2 Hz, 1H), 8.50 (dd, J=8.5, 2.3 Hz, 1H), 8.35 (s, 1H), 7.97 (d, J=8.5 Hz, 1H), 7.39 (d, J=8.5 Hz, 1H), 7.20 (s, 1H), 7.16 (br d, J=8.4 Hz, 1H), 4.49 (d, J=5.7 Hz, 2H), 2.37 (s, 3H). ^{13}C NMR (150 MHz d_6-DMSO) δ: 163.87, 153.37, 150.23, 148.05, 147.16, 142.59, 139.30, 138.49, 136.23, 129.37, 129.17, 122.26, 120.10 (q, J=255.2 Hz), 118.16, 112.44, 40.36, 18.64. Melting point: 201 ^{o}C.

Compound 19b (ONO-2920632 = VU6011887, *N*-(2-fluoro-4-(trifluoromethoxy)benzyl)-[1,2,4]triazolo[1,5-a]pyridine-6-carboxamide) was prepared according to the general procedures above using [1,2,4]triazolo[1,5-a]pyridine-6-carboxylic acid (1.59 g, 9.73 mmol), HATU, *N,N*-diisopropylethylamine, (2-fluoro-4-(trifluoromethoxy)phenyl)methanamine and DMF in 68% yield (2.64 g). LCMS (Method 1): Retention Time = 0.93 min; m/z $(M + 1)^+$ = 355. HRMS: Obs: 355.0805, Calcd: 355.0813 for $C_{15}H_{11}F_4N_4O_2$. ^{1}H NMR (600 MHz, d_6-DMSO) δ: 9.48 (dd, J=1.6, 0.9 Hz, 1H), 9.30 (t, J=5.6 Hz, 1H), 8.62 (s, 1H), 8.07 (dd, J=9.3, 1.7 Hz, 1H), 7.91 (dd, J=9.4, 0.7 Hz, 1H), 7.57 (t, J=8.6 Hz, 1H), 7.38 (dd, J=10.3, 1.5 Hz, 1H), 7.22 (br d, J=8.7 Hz, 1H), 4.55 (d, J=5.6 Hz, 2H). ^{13}C NMR (150 MHz d_6-DMSO) δ: 163.45, 159.92 (d, J=247.2 Hz), 155.41, 150.63, 147.74 (d, J=12.6 Hz), 131.13 (d, J=4.7 Hz), 129.51, 129.05, 125.33 (d, J=15.0 Hz), 121.37, 119.93 (q, J=255.2 Hz), 117.02 (d, J=3.5 Hz), 115.67, 109.16 (d, J=25.4 Hz), 36.47 (d, J=3.5 Hz). Melting point: 177 ^{o}C.

Characterization of ONO-2920632

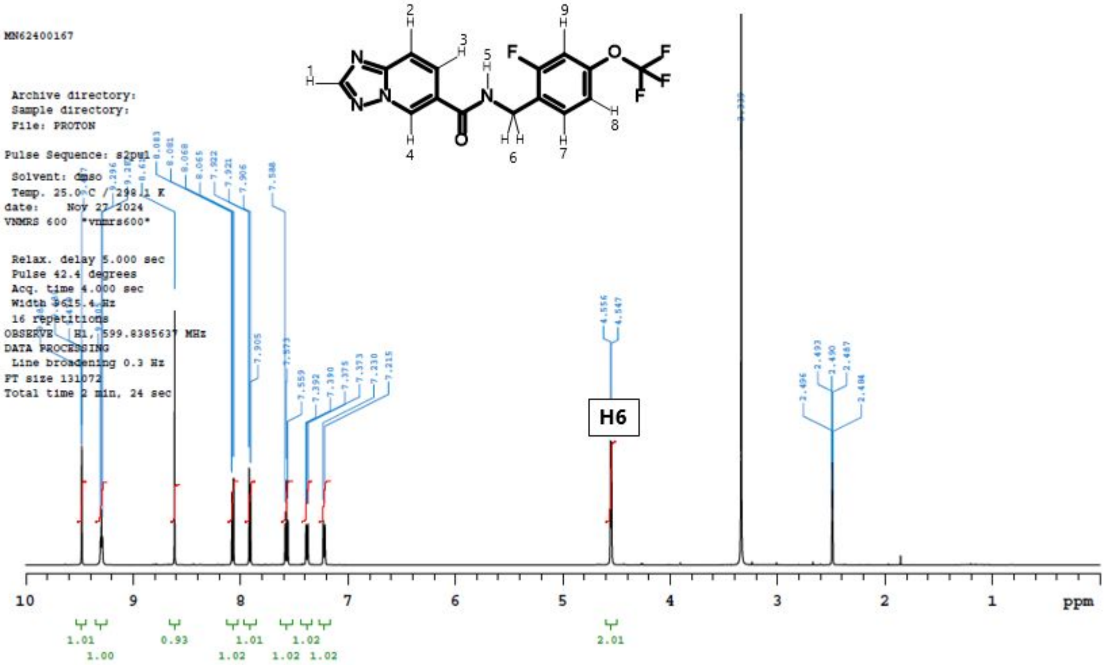

Characterization of ONO-2920632

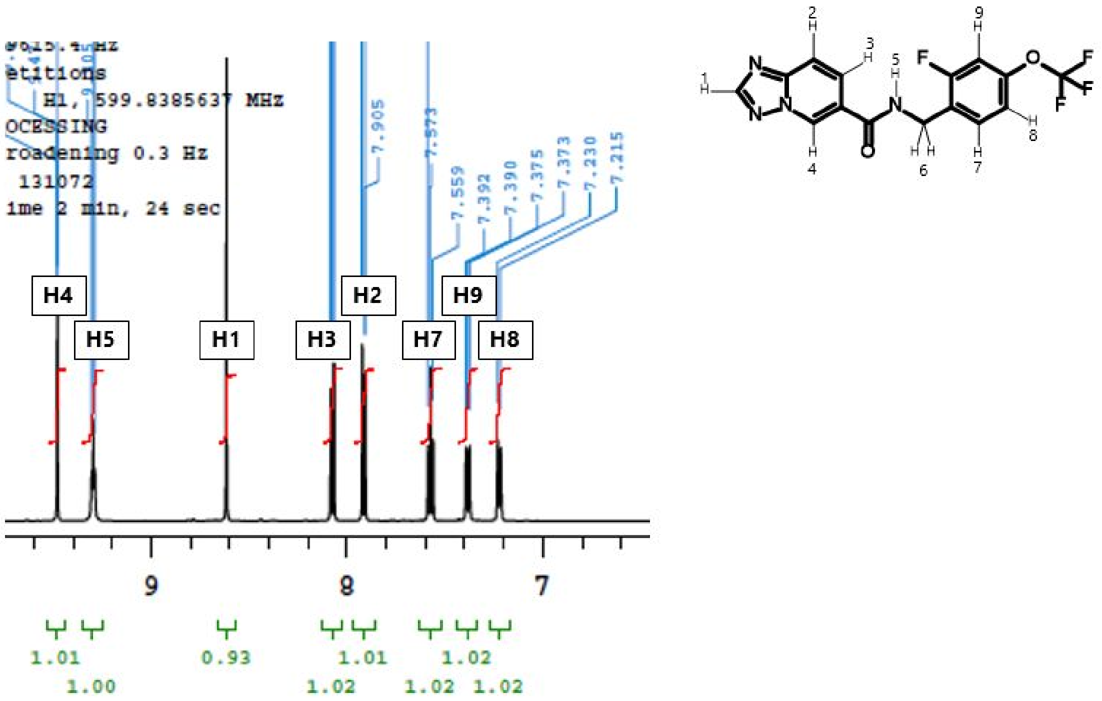

Characterization of ONO-2920632

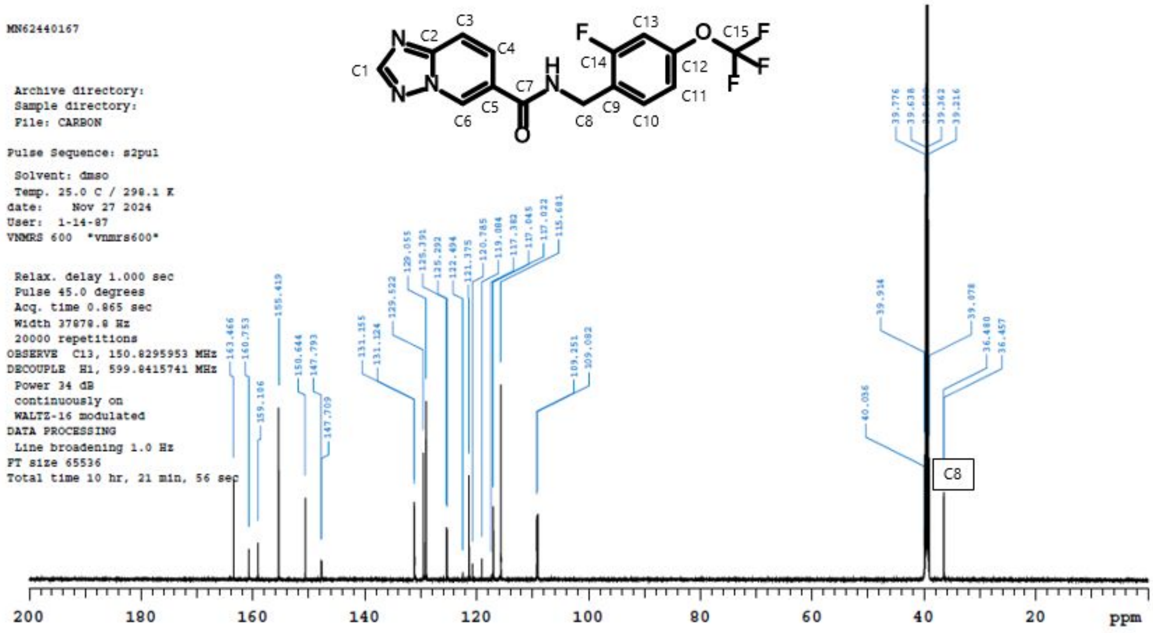

Characterization of ONO-2920632

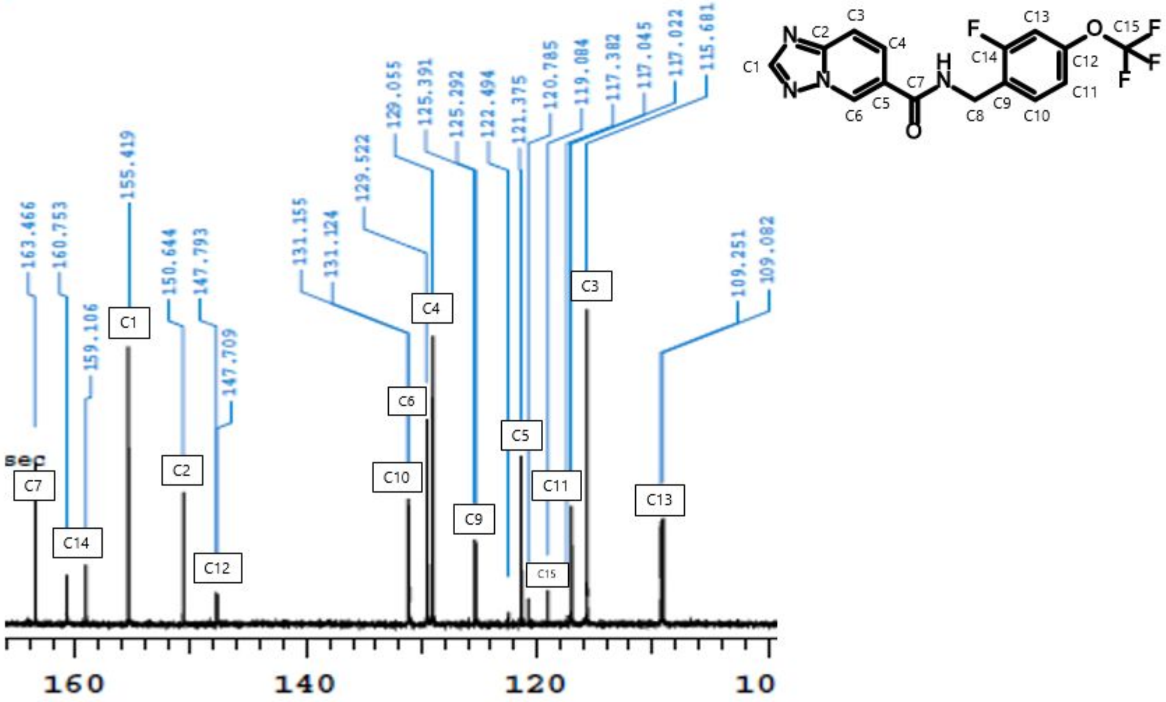

Characterization of ONO-2920632

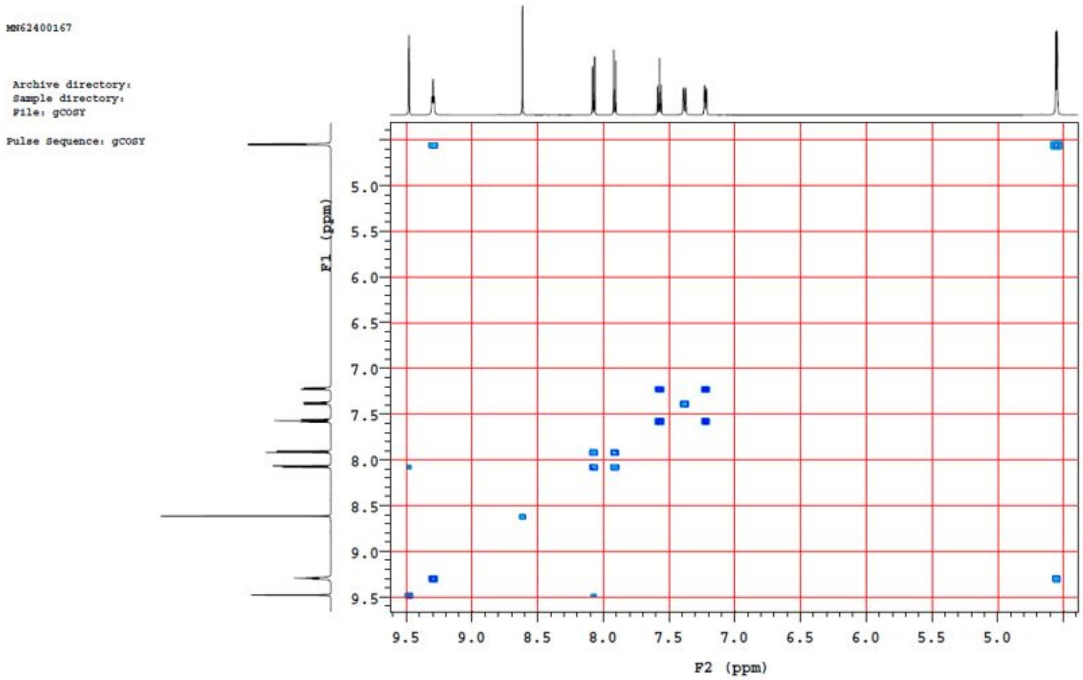

Characterization of ONO-2920632

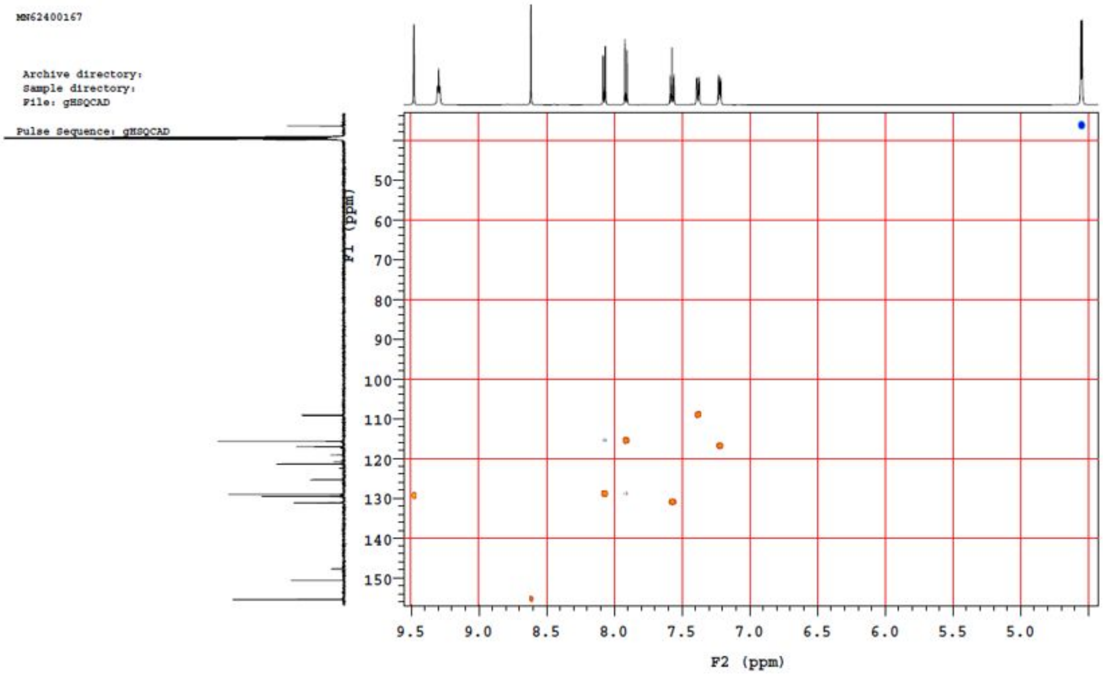

Characterization of ONO-2920632

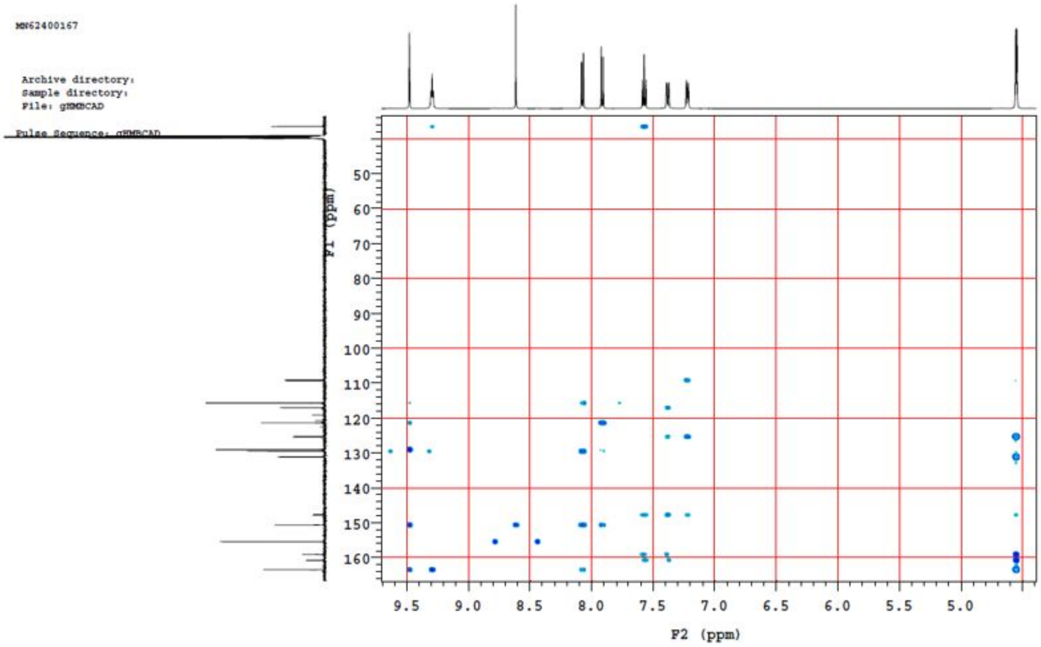

Characterization of ONO-2920632

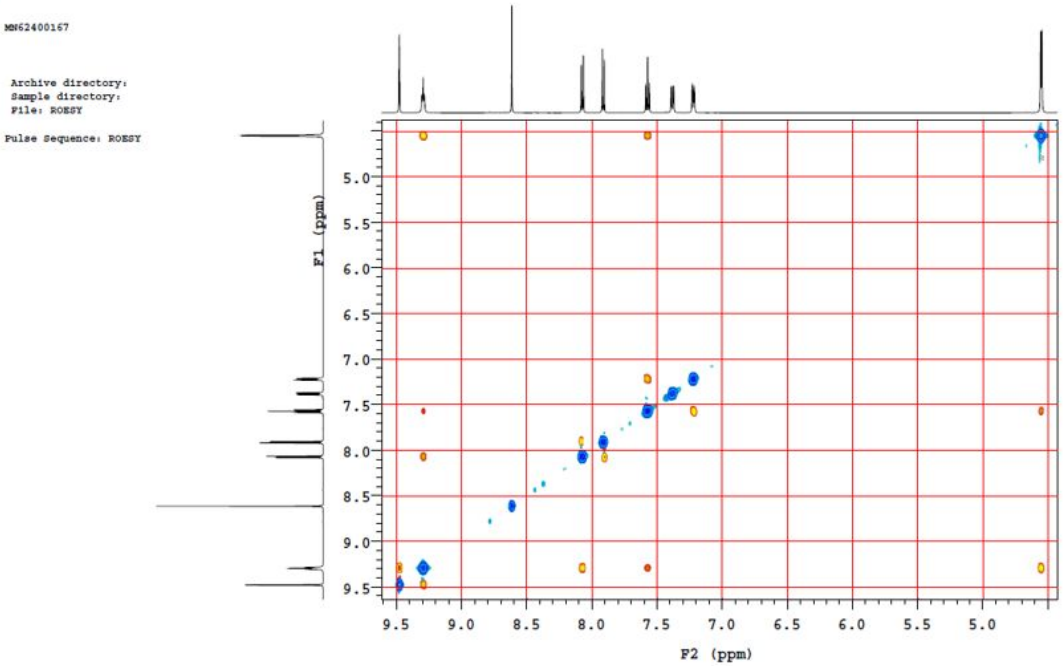

Characterization of ONO-2920632

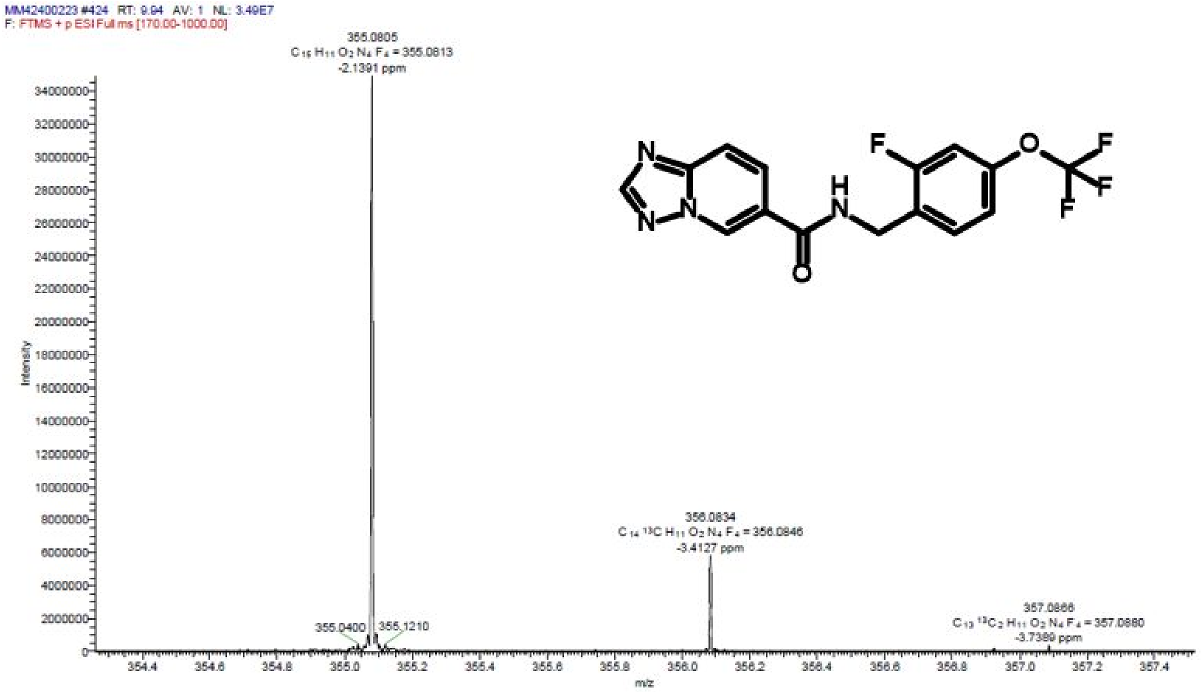

Obs: 355.0805, Calcd: 355.0813 for C15H11F4N4O2

Characterization of ONO-2950632

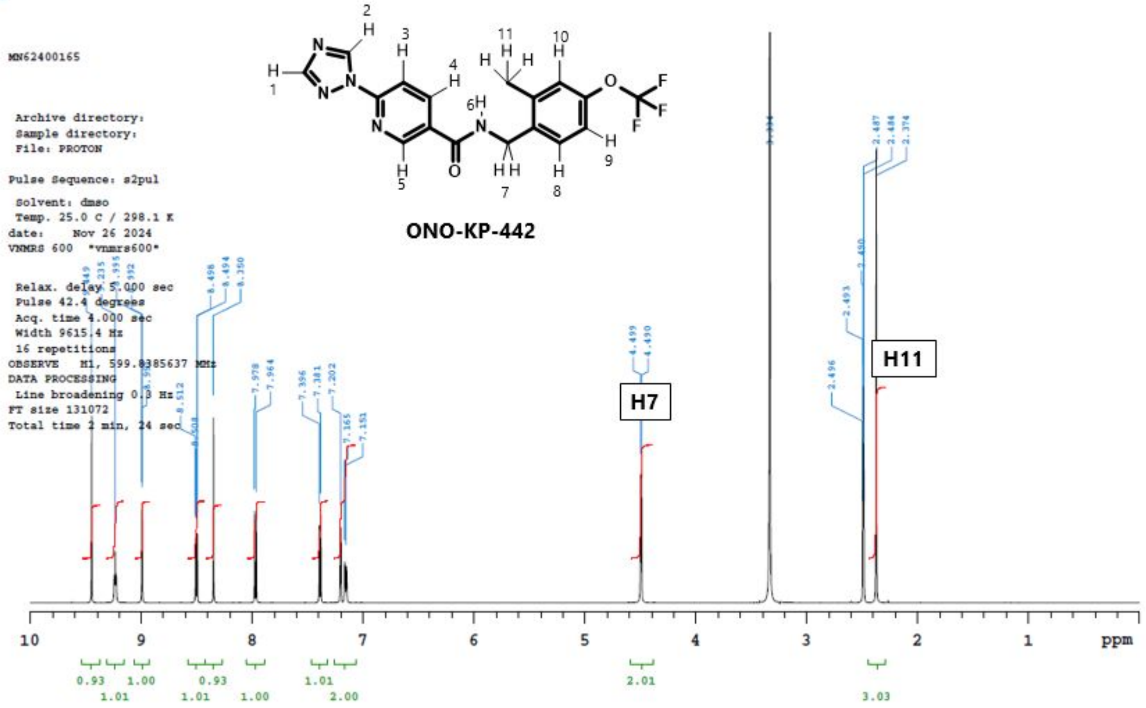

Characterization of ONO-2950632

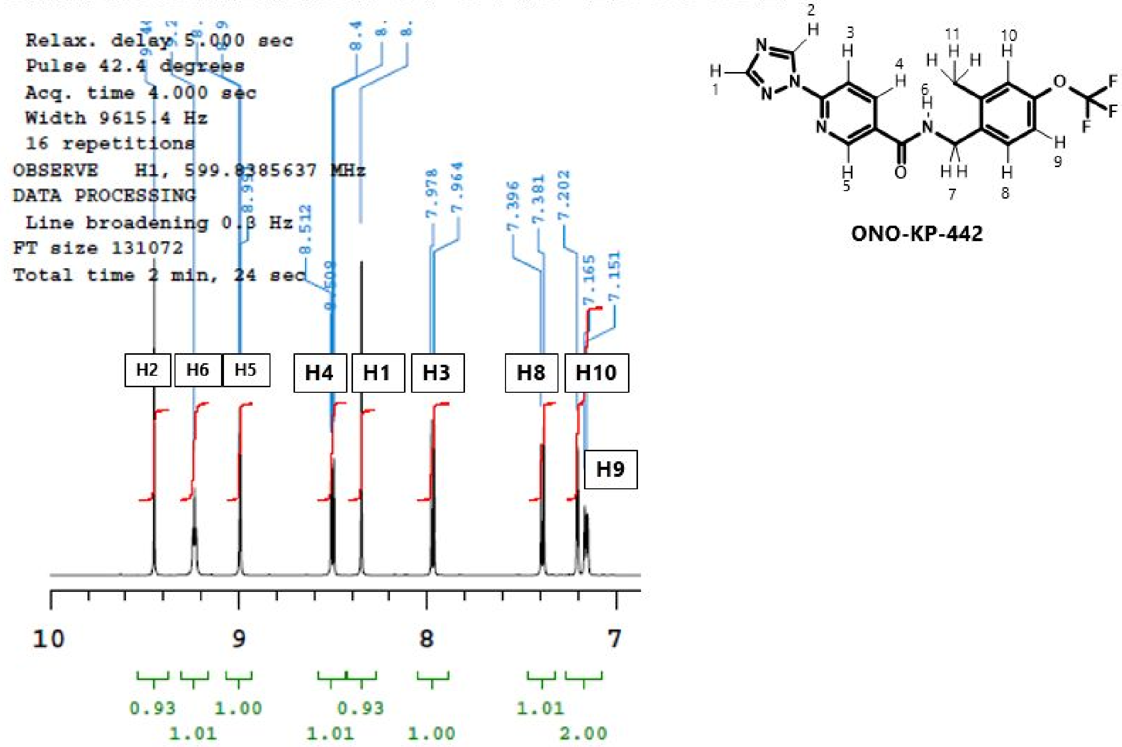

Characterization of ONO-2950632

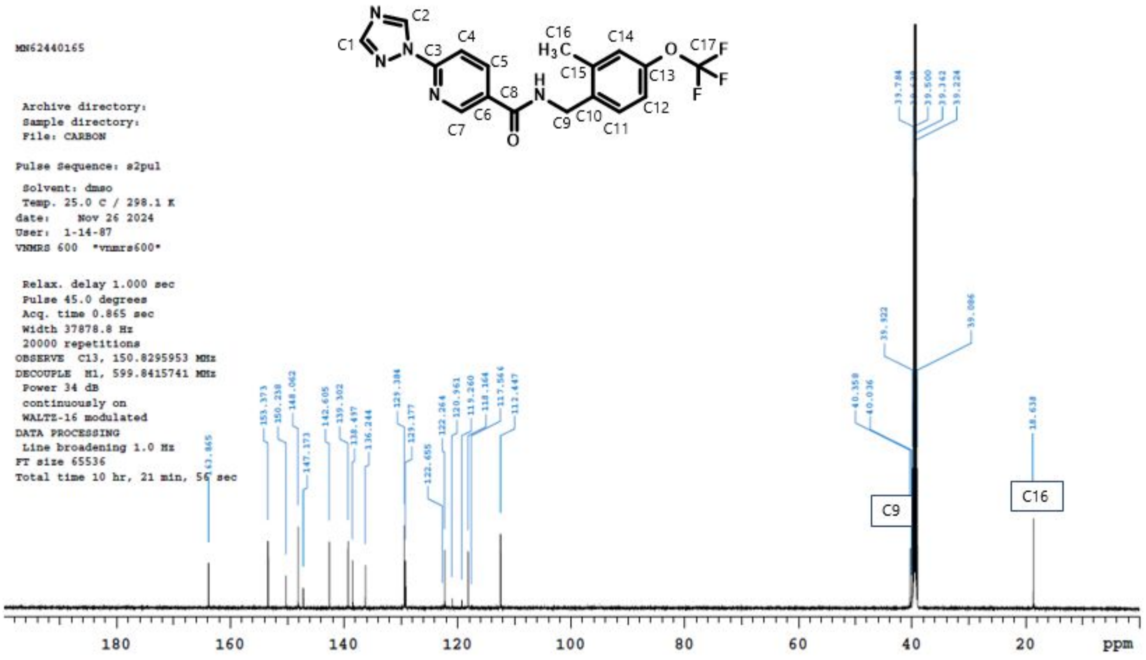

Characterization of ONO-2950632

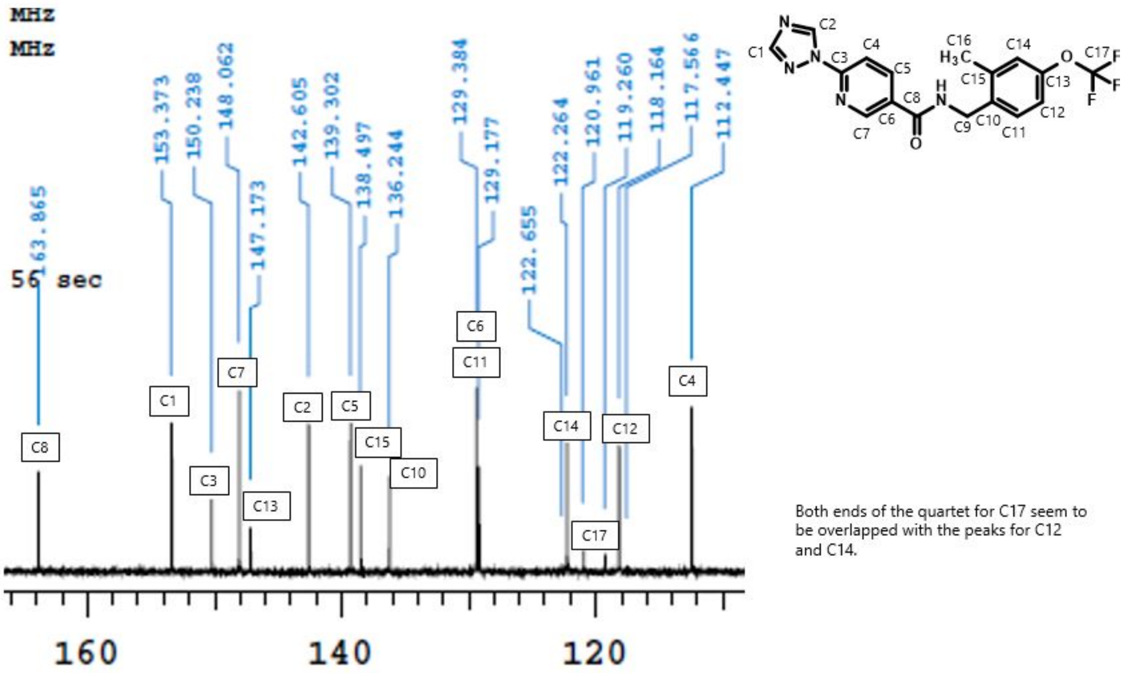

Both ends of the quartet for C17 seem to be overlapped with the peaks for C12 and C14.

Characterization of ONO-2950632

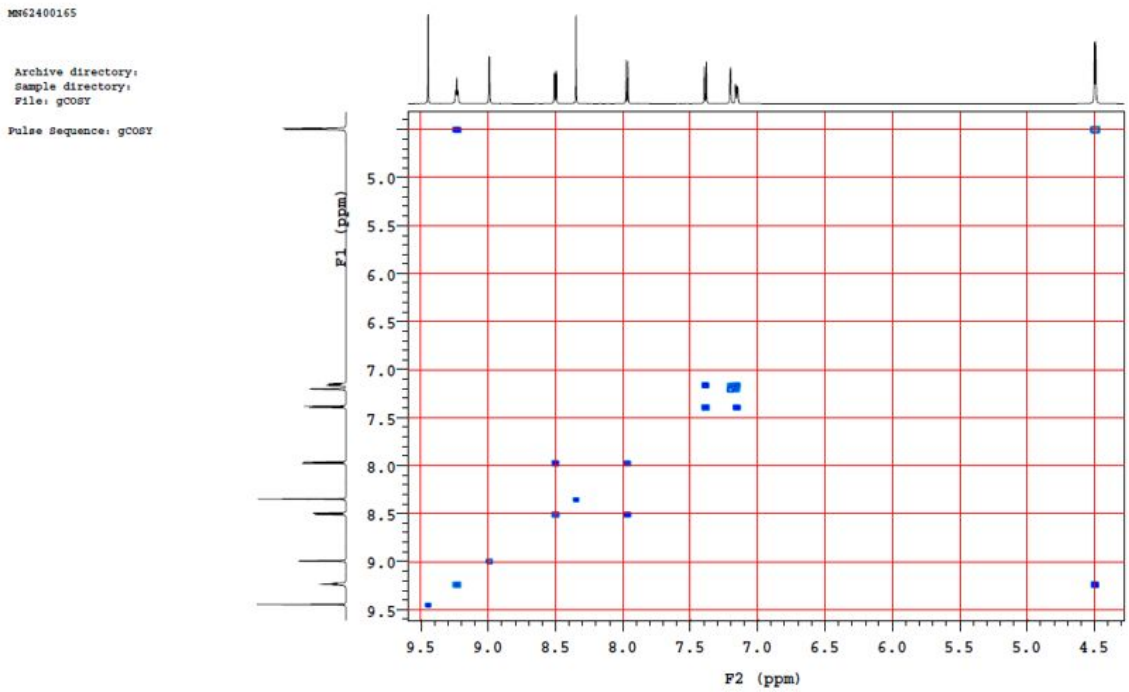

Characterization of ONO-2950632

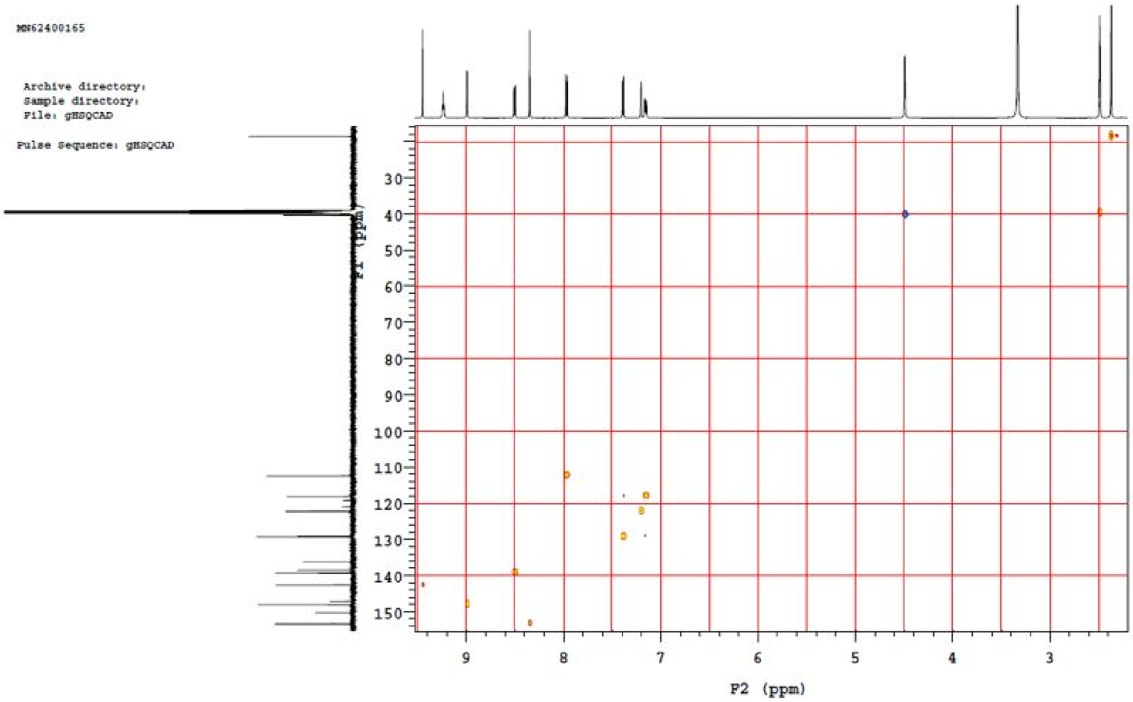

Characterization of ONO-2950632

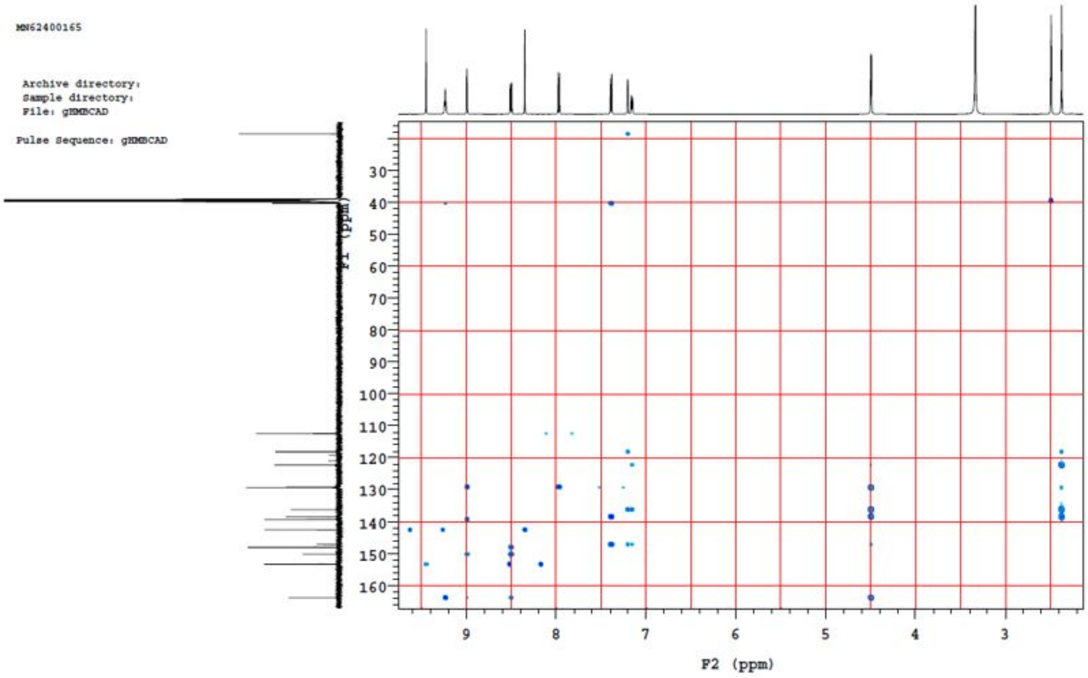

Characterization of ONO-2950632

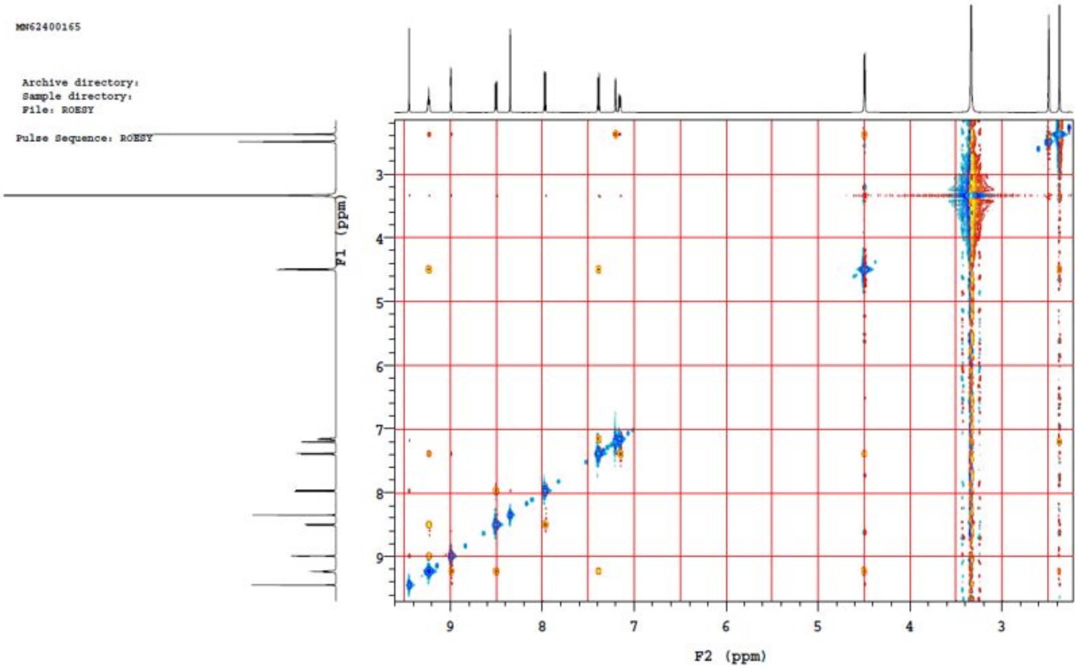

Characterization of ONO-2950632

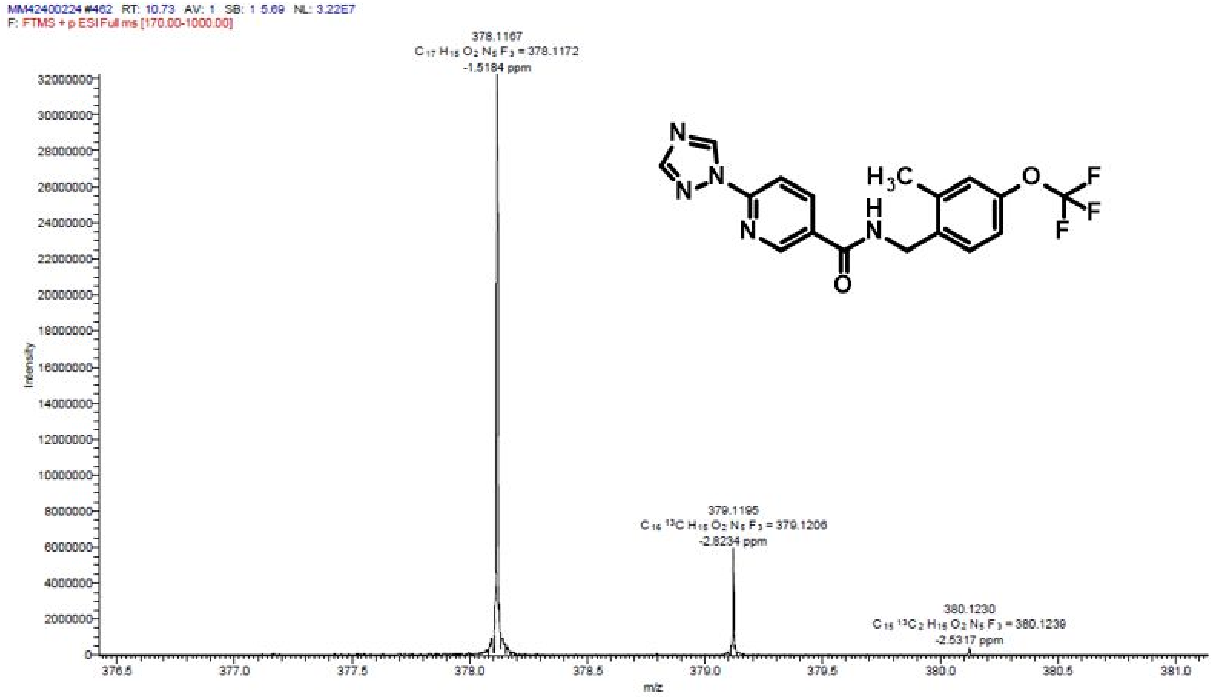

Obs: 378.1167, Calcd: 378.1172 for C17H15F3N5O2

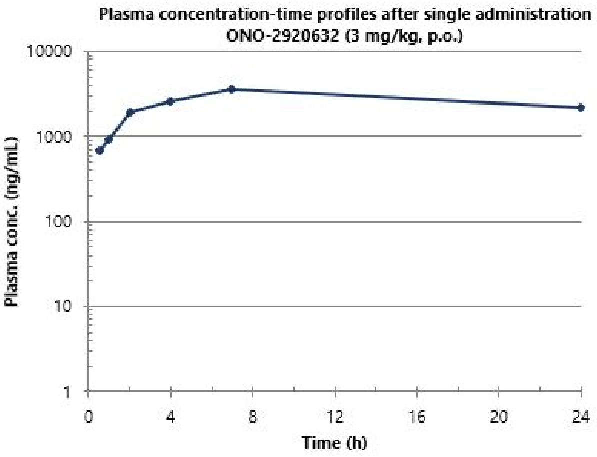

Pharmacokinetic parameters				
ONO No.		ONO-2920632		
Species		Rat		
Strains		SD		
M/F		M		
Dose	(mg/kg)	3		
Food		Non-fasted		
Route		po		
Single/cassette		single		
N		2		
Vehicle		MC		
Solution/suspension		Suspension		
Cmax	(ng/mL)	3660	±	NA
Tmax	(h)	7.0	±	NA
AUClast	(ng·h/mL)	64900	±	NA
AUCall	(ng·h/mL)	64900	±	NA
AUCinf	(ng·h/mL)	141000	±	NA
T1/2	(h)	24	±	NA
CL	(mL/min/kg)			
Vss	(mL/kg)			
BA	(%)			
Comment				

Plasma conc.(2h) (ng/mL)		CSF conc.(2h) (ng/mL)		Brain conc.(2h) (ng/g)		Brain conc.(24h) (ng/g)	
#1	#2	#1	#2	#1	#2	#1	#2
1600	3720	87.5	85.2	620	1280	1110	941

Kp (B/P, 2h): 0.37 ((0.388 + 0.344) / 2)
Kp,uu (B/P, 2h): 0.38 (fu,p: 7.50, fu,b: 7.69)

Figure S1. PK data of ONO-2920632 (rat, p.o.).

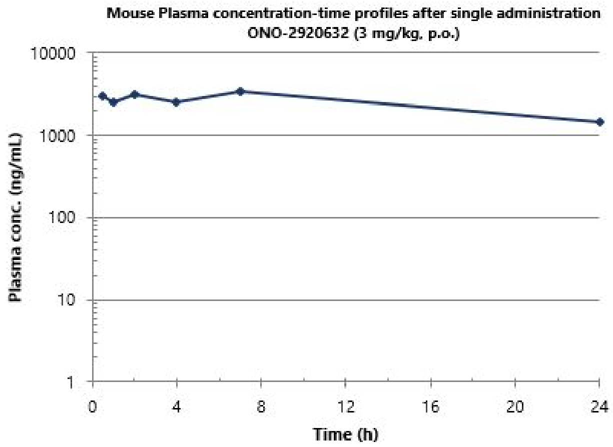

	plasma	brain	CSF	B/P
N	2hr (ng/mL)	2hr (ng/mL)	2hr (ng/mL)	2hr
1	2380	1110	83.6	0.466387
2	2890	1390	-	0.480969
3	2600	1190	70.1	0.457692
4	2160	931	97	0.431019
5	2490	1110	64.7	0.445783
Ave.	2504	1146	78.9	0.45637

Kp (B/P, 2h): 0.46
Kp,uu (B/P, 2h): 0.15 (fu,p: 24.1, fu,b: 7.69)

Pharmacokinetic parameters				
ONO No.		ONO-2920632		
Species		Mouse		
Strains		C57BL/6		
M/F		M		
Dose	(mg/kg)	3		
Food		Non-fasted		
Route		po		
Single/cassette		single		
N		2		
Vehicle		MC		
Solution/suspension		Suspension		
Cmax	(ng/mL)	3720	±	NA
Tmax	(h)	3.8	±	NA
AUClast	(ng·h/mL)	59000	±	NA
AUCall	(ng·h/mL)	59000	±	NA
AUCinf	(ng·h/mL)	100000	±	NA
T1/2	(h)	19	±	NA
CL	(mL/min/kg)			
Vss	(mL/kg)			
BA	(%)			
Comment				

Figure S2. PK data of ONO-2920632 (mouse, p.o.).

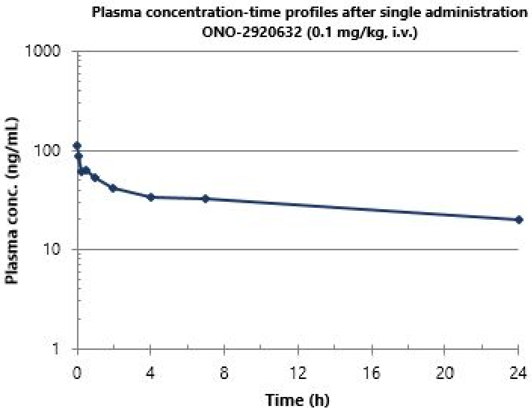

Pharmacokinetic parameters				
ONO No.		ONO-2920632		
Species		Dog		
Strains		beagle		
M/F		M		
Dose	(mg/kg)	0.1		
Food		fasted		
Route		iv		
Single/cassette		Single		
N		2		
Vehicle		HP-b-CD		
Solution/suspension		Solution		
Cmax	(ng/mL)			
Tmax	(h)			
AUClast	(ng·h/mL)	728	±	NA
AUCall	(ng·h/mL)	728	±	NA
AUCinf	(ng·h/mL)	1390	±	NA
T1/2	(h)	22	±	NA
CL	(mL/min/kg)	1.29	±	NA
Vss	(mL/kg)	2230	±	NA
BA	(%)			
Comment				

Figure S3. PK data of ONO-2920632 (dog, i.v.).

The analgesic effect in acetic acid writhing model (ICR mouse)

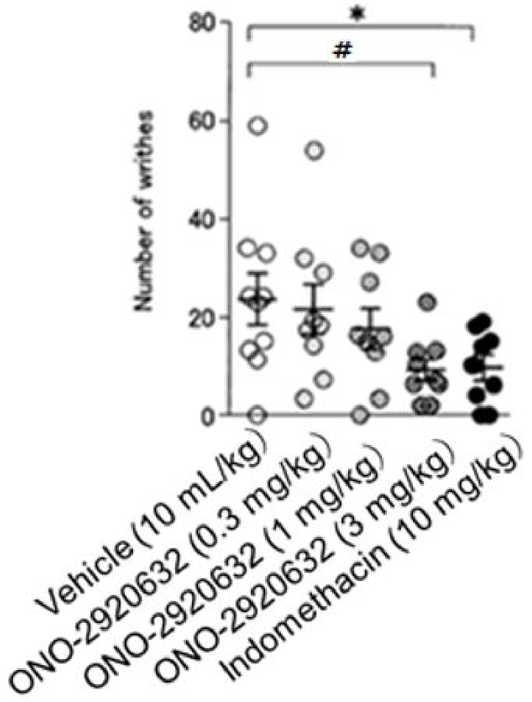

Plasma conc (μM) at 2.5hr after oral dosing (Just after counting the number of writhes on the model)

N	0.3 mg/kg	1 mg/kg	3 mg/kg
1	0.27	1.70	3.61
2	0.29	1.28	3.61
3	0.23	1.23	2.64
4	0.29	1.43	3.16
5	0.31	1.71	
Average	0.28	1.47	3.26

Figure S4. In vivo efficacy data of ONO-2920632.

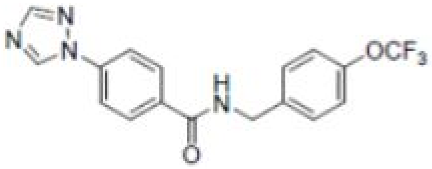

ONO-E0005-383

TREK-1 IC50 = 4.4 uM (68%)
TREK-2 IC50 = 1.2 uM (156%)

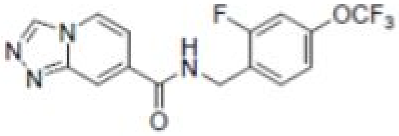

ONO-KP-191

TREK-1 IC50 = 2.9 uM (96%)
TREK-2 IC50 = 0.28 uM (185%)

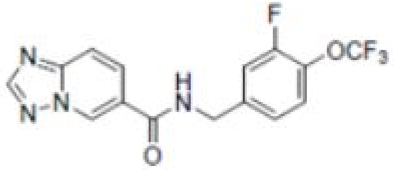

ONO-KP-181

TREK-1 IC50 = 6.7 uM (88%)
TREK-2 IC50 = 1.3 uM (118%)

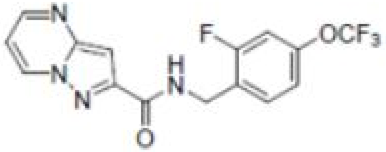

ONO-KP-341

TREK-1 IC50 = 3.4 uM (49%)
TREK-2 IC50 = 1.2 uM (153%)

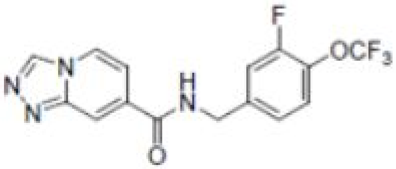

ONO-KP-185

TREK-1 IC50 = 11.1 uM (61%)
TREK-2 IC50 = 5.6 uM (141%)

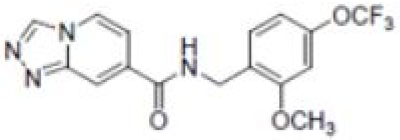

ONO-KP-192

TREK-1 IC50 = 10.8 uM (76%)
TREK-2 IC50 = 0.35 uM (152%)

Figure S5. Additional TREK SAR
